# Supplementary material for: Evaluation of 16S rRNA gene sequencing for species and strain-level microbiome analysis
Source: Nat Commun. 2019 Nov 6;10:5029. doi: 10.1038/s41467-019-13036-1 (PMC6834636; doi:10.1038/s41467-019-13036-1)
Supplement: Supplementary file 1 — Supplementary Information [file 41467_2019_13036_MOESM1_ESM.pdf]

Supplementary Information

Evaluation of 16S rRNA gene sequencing for species and strain-level microbiome analysis

Johnson et al.

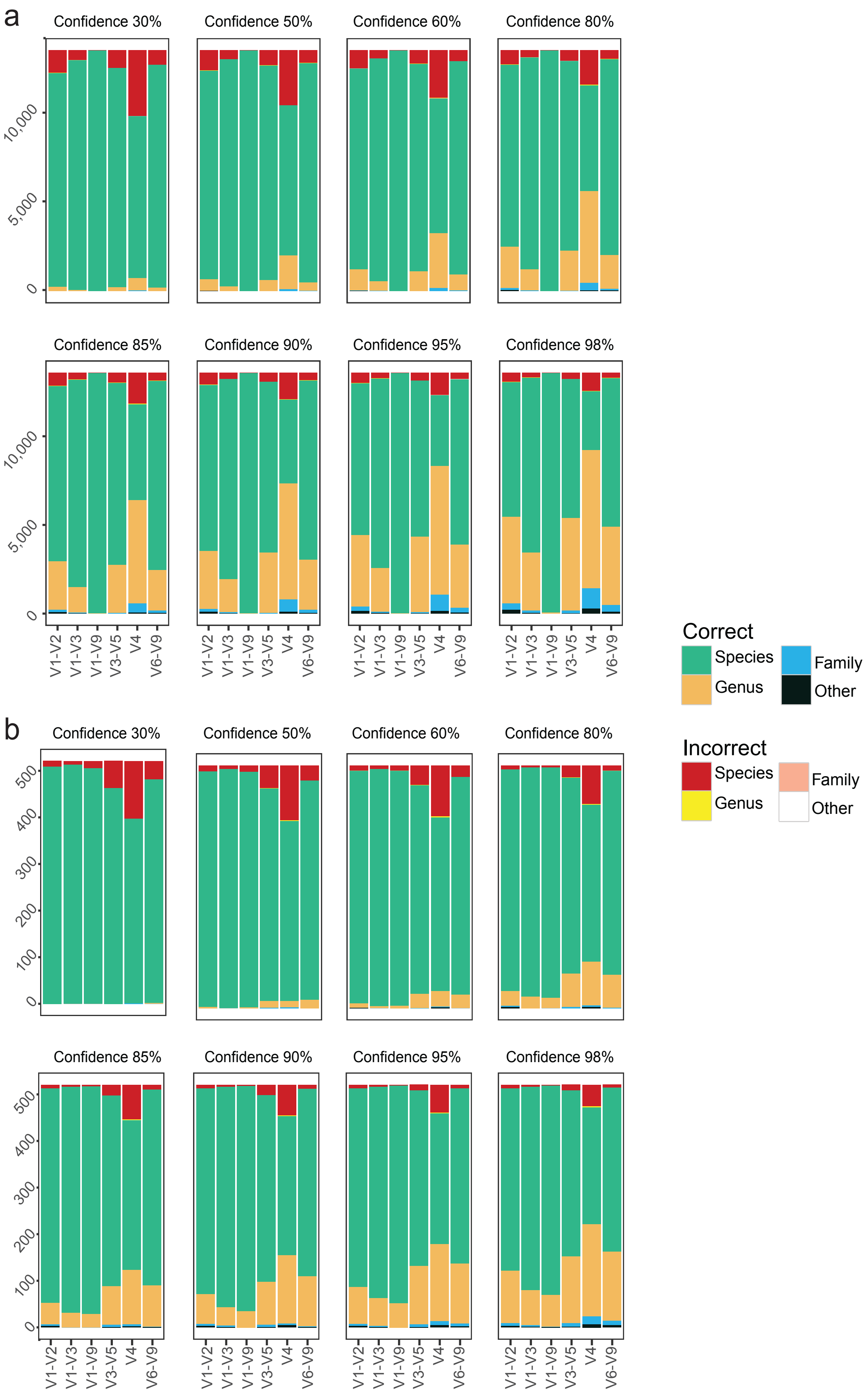

Supplementary Figure 1: The success with which *in silico* amplicons can be reclassified to the correct species, shown for a) Greengenes and b) HOMD databases. The RDP classifier returns a lowest possible taxonomic prediction for a given confidence threshold. Stacked bar charts indicate both the taxonomic level at which a prediction was returned and whether that prediction was correct. Source data are provided as a Source Data file.

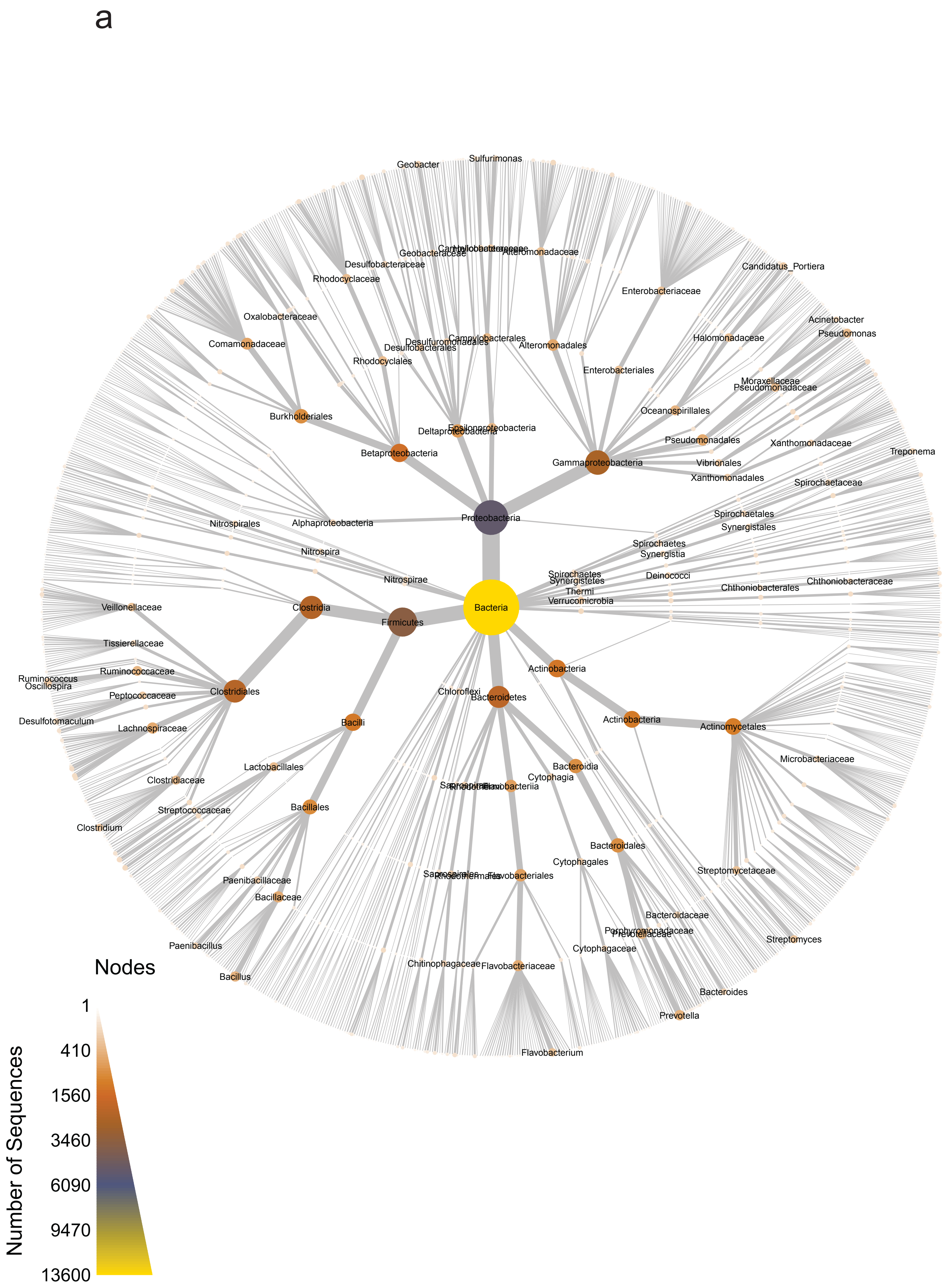
a
Bacteria
Proteobacteria
Firmicutes
Betaproteobacteria
Gammaproteobacteria
Alphaproteobacteria
Deltaproteobacteria
Epsilonproteobacteria
Burkholderiales
Comamonadaceae
Oxalobacteraceae
Rhodocyclales
Rhodocyclaceae
Desulfobacterales
Desulfobacteraceae
Desulfuromonadales
Geobacteraceae
Geobacter
Sulfurimonas
Campylobacterales
Alteromonadales
Alteromonadaceae
Enterobacteriales
Enterobacteriaceae
Candidatus_Portiera
Halomonadaceae
Acinetobacter
Pseudomonas
Moraxellaceae
Pseudomonadaceae
Oceanospirillales
Pseudomonadales
Xanthomonadaceae
Vibrionales
Xanthomonadales
Treponema
Spirochaetaceae
Spirochaetales
Synergistales
Spirochaetes
Synergistia
Synergistetes
Deinococci
Thermi
Chthoniobacterales
Chthoniobacteraceae
Verrucomicrobia
Nitrospirales
Nitrospira
Nitrospirae
Clostridia
Veillonellaceae
Tissierellaceae
Ruminococcaceae
Ruminococcus
Oscillospira
Peptococcaceae
Clostridiales
Desulfotomaculum
Lachnospiraceae
Clostridiaceae
Clostridium
Bacilli
Lactobacillales
Streptococcaceae
Bacillales
Paenibacillaceae
Bacillaceae
Paenibacillus
Bacillus
Chloroflexi
Bacteroidetes
Actinobacteria
Actinomycetales
Microbacteriaceae
Bacteroidia
Cytophagia
Flavobacteriia
Bacteroidales
Cytophagales
Flavobacteriales
Streptomycetaceae
Bacteroidaceae
Porphyromonadaceae
Prevotellaceae
Cytophagaceae
Streptomyces
Chitinophagaceae
Flavobacteriaceae
Bacteroides
Prevotella
Flavobacterium
Nodes
Number of Sequences
1
410
1560
3460
6090
9470
13600

b

V1-V2

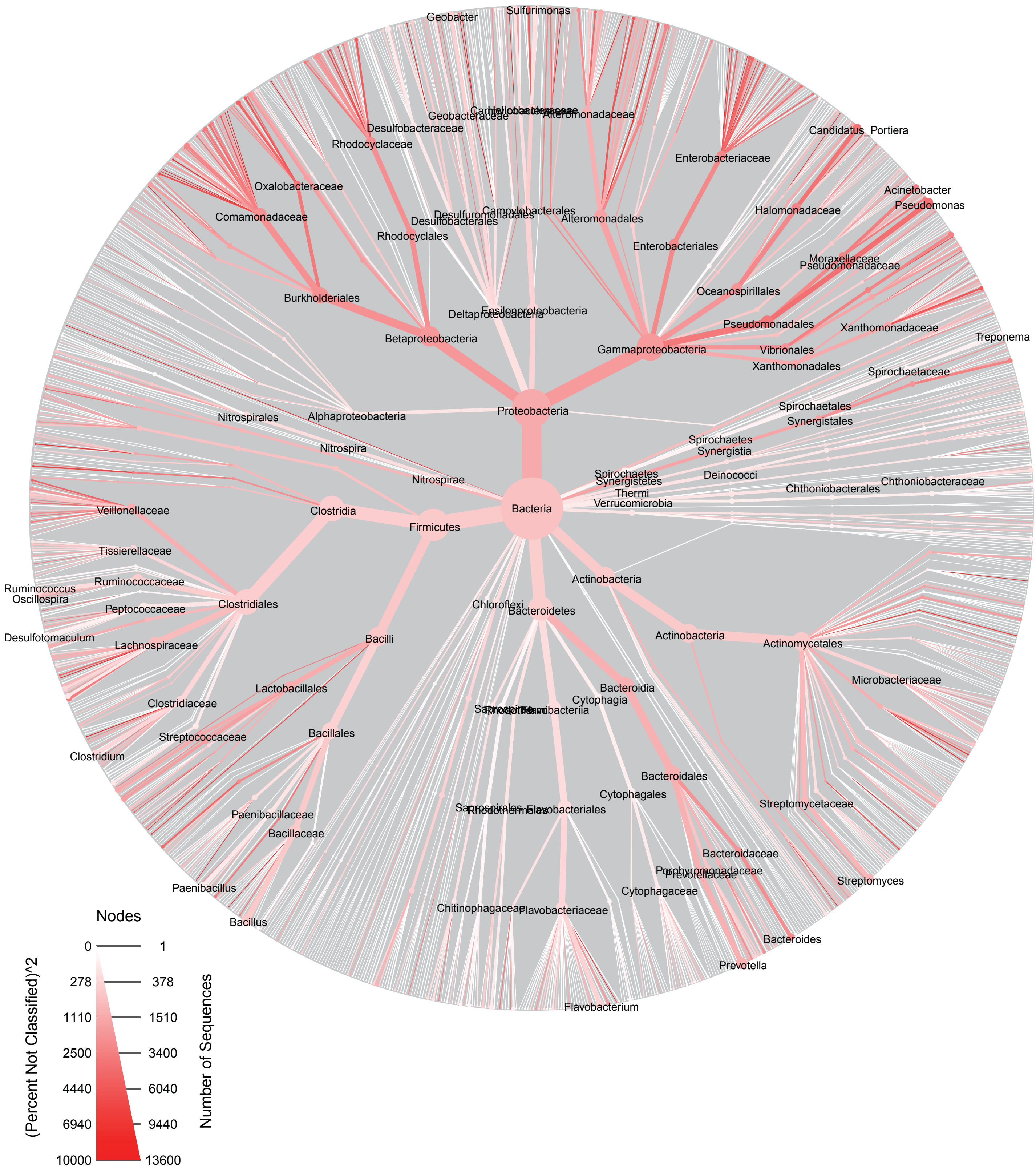

Geobacter
Sulfurimonas
Geobacteraceae
Alteromonadaceae
Desulfobacteraceae
Rhodocyclaceae
Candidatus_Portiera
Enterobacteriaceae
Oxalobacteraceae
Acinetobacter
Pseudomonas
Comamonadaceae
Campylobacterales
Desulfuromonadales
Alteromonadales
Halomonadaceae
Desulfobacterales
Rhodocyclales
Enterobacteriales
Moraxellaceae
Pseudomonadaceae
Burkholderiales
Oceanospirillales
Epsilonproteobacteria
Deltaproteobacteria
Pseudomonadales
Xanthomonadaceae
Betaproteobacteria
Treponema
Gammaproteobacteria
Vibrionales
Xanthomonadales
Spirochaetaceae
Proteobacteria
Nitrospirales
Alphaproteobacteria
Spirochaetales
Synergistales
Spirochaetes
Nitrospira
Synergistia
Deinococci
Nitrospirae
Spirochaetes
Synergistetes
Chthoniobacterales
Chthoniobacteraceae
Thermi
Verrucomicrobia
Bacteria
Veillonellaceae
Clostridia
Firmicutes
Tissierellaceae
Actinobacteria
Ruminococcaceae
Ruminococcus
Oscillospira
Clostridiales
Chloroflexi
Bacteroidetes
Peptococcaceae
Desulfotomaculum
Bacilli
Actinobacteria
Actinomycetales
Lachnospiraceae
Microbacteriaceae
Lactobacillales
Bacteroidia
Cytophagia
Clostridiaceae
Rhodothermi
Flavobacteriia
Bacillales
Streptococcaceae
Clostridium
Bacteroidales
Cytophagales
Streptomycetaceae
Paenibacillaceae
Rhodothermales
Flavobacteriales
Bacillaceae
Bacteroidaceae
Porphyromonadaceae
Prevotellaceae
Streptomyces
Paenibacillus
Cytophagaceae
Chitinophagaceae
Flavobacteriaceae
Nodes
Bacillus
Bacteroides
Prevotella
Flavobacterium
0
1
278
378
1110
1510
2500
3400
4440
6040
6940
9440
10000
13600
(Percent Not Classified)^2
Number of Sequences

c

V1-V3

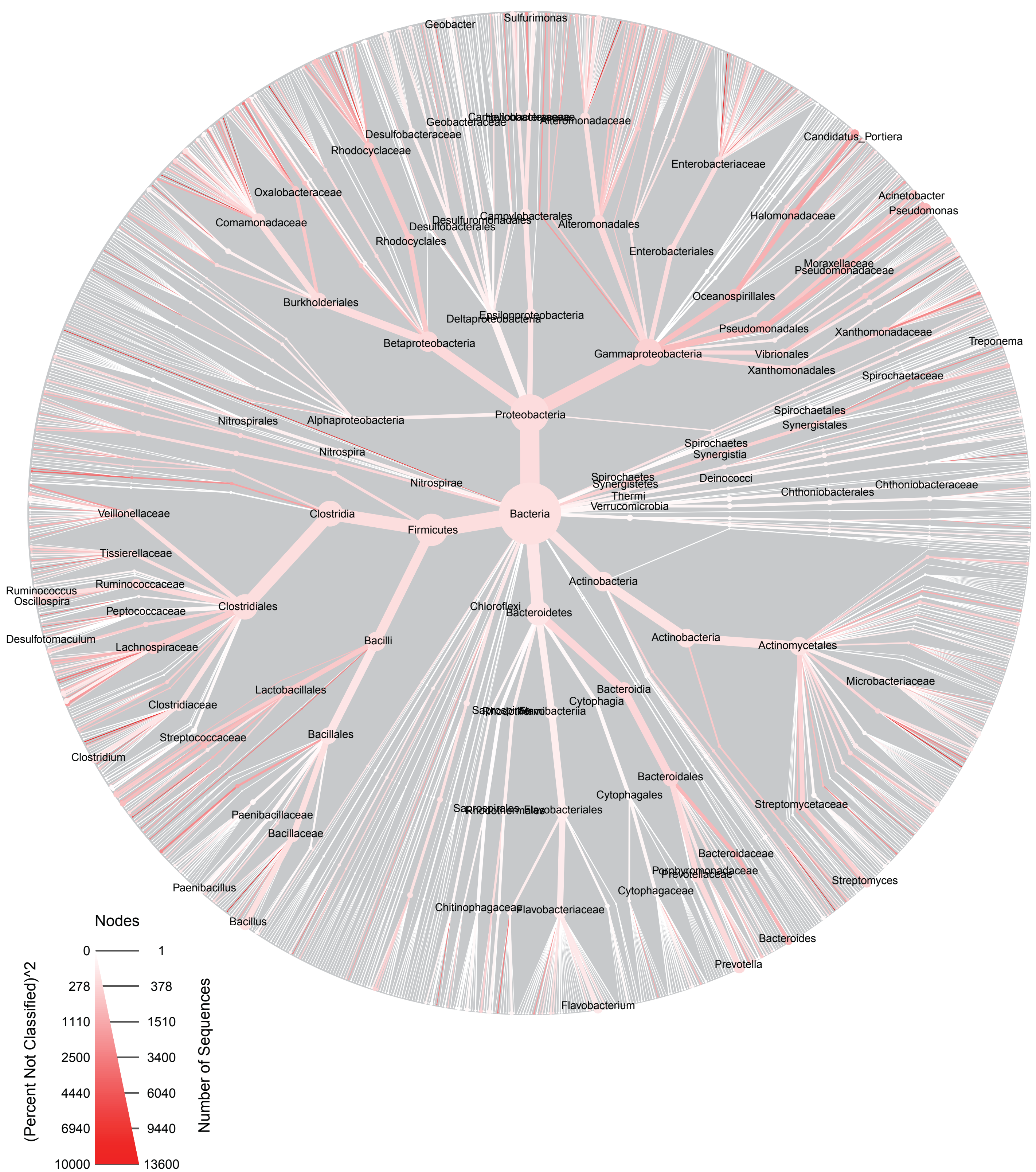

Bacteria
Proteobacteria
Gammaproteobacteria
Betaproteobacteria
Alphaproteobacteria
Deltaproteobacteria
Epsilonproteobacteria
Burkholderiales
Comamonadaceae
Oxalobacteraceae
Rhodocyclales
Rhodocyclaceae
Desulfobacterales
Desulfobacteraceae
Desulfuromonadales
Geobacteraceae
Geobacter
Sulfurimonas
Campylobacterales
Alteromonadales
Alteromonadaceae
Enterobacteriales
Enterobacteriaceae
Candidatus_Portiera
Halomonadaceae
Acinetobacter
Pseudomonas
Oceanospirillales
Moraxellaceae
Pseudomonadaceae
Pseudomonadales
Xanthomonadaceae
Vibrionales
Xanthomonadales
Treponema
Spirochaetaceae
Spirochaetales
Synergistales
Spirochaetes
Synergistia
Deinococci
Chthoniobacterales
Chthoniobacteraceae
Synergistetes
Thermi
Verrucomicrobia
Nitrospirales
Nitrospira
Nitrospirae
Clostridia
Firmicutes
Veillonellaceae
Tissierellaceae
Ruminococcaceae
Ruminococcus
Oscillospira
Clostridiales
Peptococcaceae
Desulfotomaculum
Lachnospiraceae
Bacilli
Lactobacillales
Clostridiaceae
Streptococcaceae
Clostridium
Bacillales
Paenibacillaceae
Bacillaceae
Paenibacillus
Bacillus
Chloroflexi
Bacteroidetes
Actinobacteria
Actinomycetales
Microbacteriaceae
Bacteroidia
Cytophagia
Flavobacteriia
Bacteroidales
Cytophagales
Flavobacteriales
Streptomycetaceae
Bacteroidaceae
Porphyromonadaceae
Prevotellaceae
Streptomyces
Cytophagaceae
Chitinophagaceae
Flavobacteriaceae
Bacteroides
Prevotella
Flavobacterium
Nodes
(Percent Not Classified)^2
0
278
1110
2500
4440
6940
10000
Number of Sequences
1
378
1510
3400
6040
9440
13600

d

V1-V9

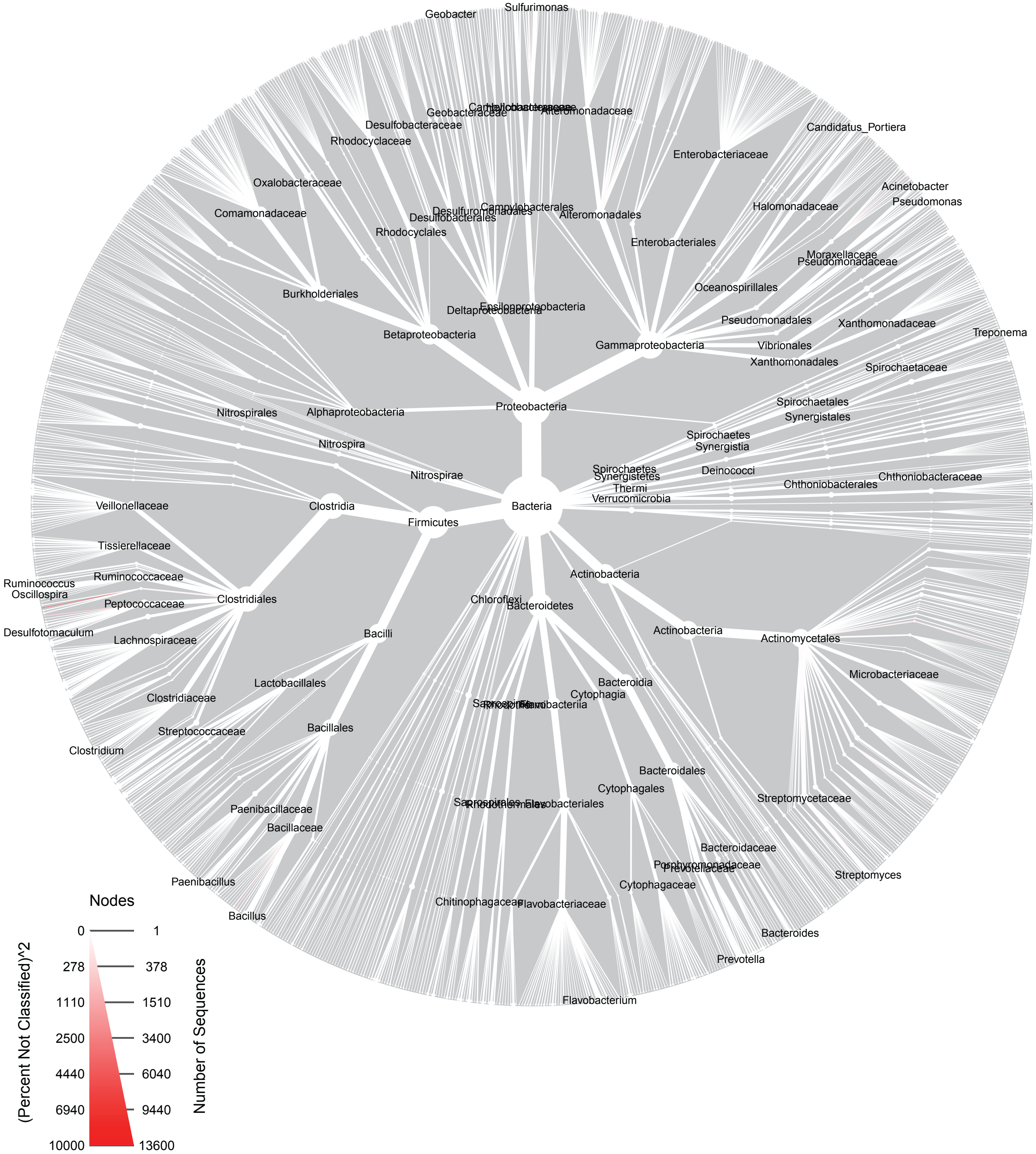

Geobacter
Sulfurimonas
Geobacteraceae
Alteromonadaceae
Desulfobacteraceae
Rhodocyclaceae
Candidatus_Portiera
Enterobacteriaceae
Oxalobacteraceae
Acinetobacter
Pseudomonas
Comamonadaceae
Halomonadaceae
Campylobacterales
Desulfobacterales
Alteromonadales
Rhodocyclales
Enterobacteriales
Moraxellaceae
Pseudomonadaceae
Oceanospirillales
Burkholderiales
Pseudomonadales
Xanthomonadaceae
Betaproteobacteria
Gammaproteobacteria
Vibrionales
Treponema
Xanthomonadales
Spirochaetaceae
Spirochaetales
Nitrospirales
Alphaproteobacteria
Proteobacteria
Synergistales
Spirochaetes
Nitrospira
Synergistia
Deinococci
Nitrospirae
Chthoniobacterales
Chthoniobacteraceae
Thermi
Verrucomicrobia
Veillonellaceae
Clostridia
Bacteria
Firmicutes
Tissierellaceae
Actinobacteria
Ruminococcus
Ruminococcaceae
Oscillospira
Clostridiales
Chloroflexi
Bacteroidetes
Peptococcaceae
Desulfotomaculum
Bacilli
Lachnospiraceae
Actinomycetales
Microbacteriaceae
Lactobacillales
Bacteroidia
Cytophagia
Clostridiaceae
Streptococcaceae
Bacillales
Clostridium
Bacteroidales
Cytophagales
Streptomycetaceae
Paenibacillaceae
Flavobacteriales
Bacillaceae
Bacteroidaceae
Prevotellaceae
Streptomyces
Paenibacillus
Cytophagaceae
Chitinophagaceae
Flavobacteriaceae
Bacillus
Nodes
0
1
278
378
1110
1510
2500
3400
4440
6040
6940
9440
10000
13600
(Percent Not Classified)^2
Number of Sequences
Bacteroides
Prevotella
Flavobacterium

e

V3-V5

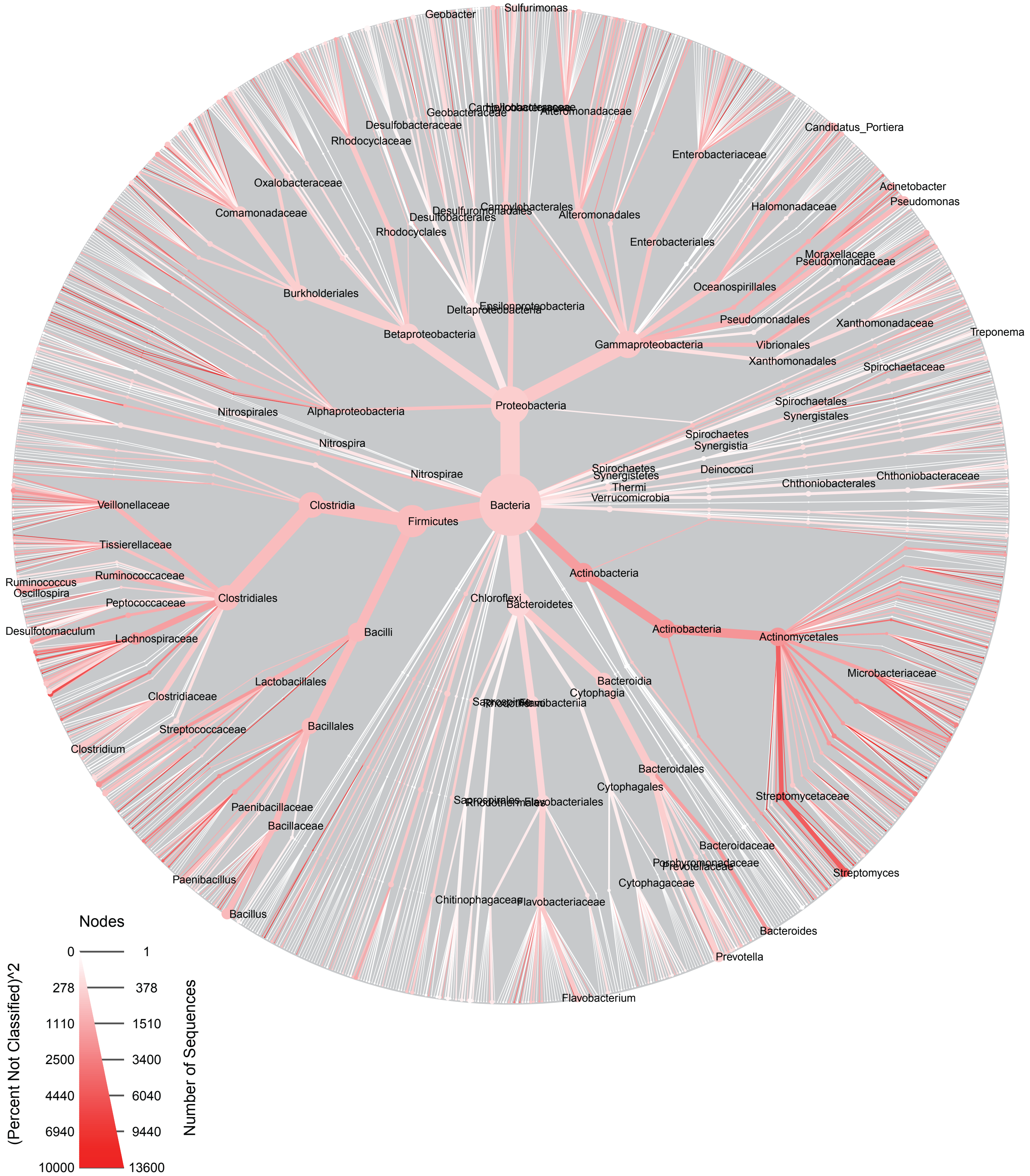
Sulfurimonas
Geobacter
Geobacteraceae
Alteromonadaceae
Desulfobacteraceae
Rhodocyclaceae
Candidatus_Portiera
Enterobacteriaceae
Oxalobacteraceae
Acinetobacter
Pseudomonas
Comamonadaceae
Campylobacterales
Alteromonadales
Halomonadaceae
Desulfobacterales
Rhodocyclales
Enterobacteriales
Moraxellaceae
Pseudomonadaceae
Oceanospirillales
Burkholderiales
Deltaproteobacteria
Epsilonproteobacteria
Pseudomonadales
Xanthomonadaceae
Betaproteobacteria
Gammaproteobacteria
Vibrionales
Treponema
Xanthomonadales
Spirochaetaceae
Proteobacteria
Nitrospirales
Alphaproteobacteria
Spirochaetales
Synergistales
Spirochaetes
Nitrospira
Synergistia
Nitrospirae
Spirochaetes
Synergistetes
Deinococci
Chthoniobacteraceae
Chthoniobacterales
Thermi
Verrucomicrobia
Veillonellaceae
Clostridia
Bacteria
Firmicutes
Tissierellaceae
Actinobacteria
Ruminococcaceae
Ruminococcus
Oscillospira
Clostridiales
Chloroflexi
Bacteroidetes
Peptococcaceae
Actinobacteria
Actinomycetales
Desulfotomaculum
Bacilli
Lachnospiraceae
Microbacteriaceae
Bacteroidia
Lactobacillales
Cytophagia
Clostridiaceae
Streptococcaceae
Bacillales
Clostridium
Bacteroidales
Cytophagales
Streptomycetaceae
Paenibacillaceae
Flavobacteriales
Bacillaceae
Bacteroidaceae
Porphyromonadaceae
Prevotellaceae
Paenibacillus
Cytophagaceae
Streptomyces
Chitinophagaceae
Flavobacteriaceae
Bacillus
Bacteroides
Prevotella
Flavobacterium
Nodes
0
1
278
378
1110
1510
2500
3400
4440
6040
6940
9440
10000
13600
(Percent Not Classified)^2
Number of Sequences

f

V4

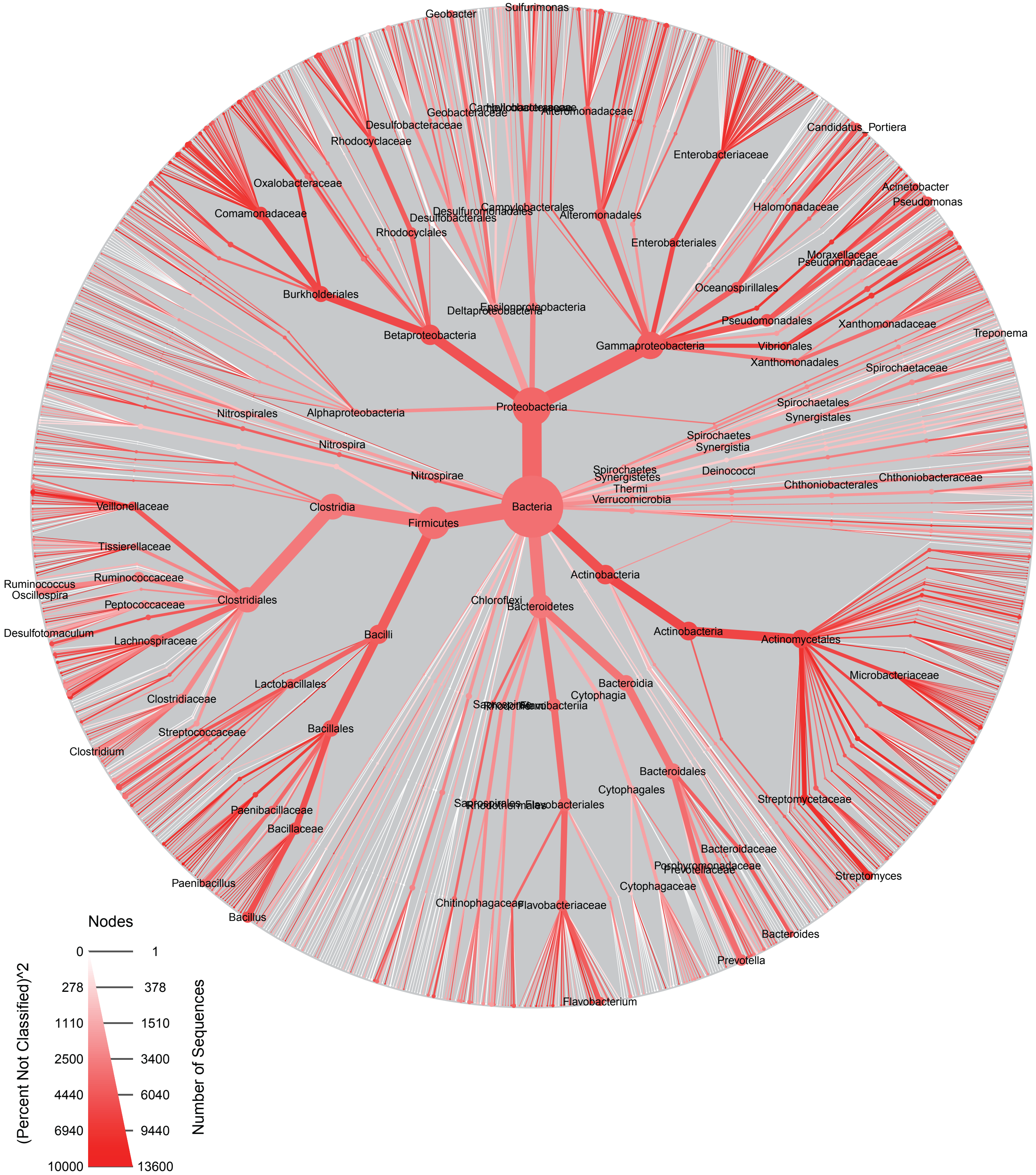
Sulfurimonas
Geobacter
Geobacteraceae
Alteromonadaceae
Desulfobacteraceae
Candidatus_Portiera
Rhodocyclaceae
Enterobacteriaceae
Oxalobacteraceae
Acinetobacter
Pseudomonas
Halomonadaceae
Comamonadaceae
Campylobacterales
Desulfobacterales
Alteromonadales
Rhodocyclales
Enterobacteriales
Moraxellaceae
Pseudomonadaceae
Oceanospirillales
Burkholderiales
Deltaproteobacteria
Pseudomonadales
Xanthomonadaceae
Betaproteobacteria
Treponema
Gammaproteobacteria
Vibrionales
Xanthomonadales
Spirochaetaceae
Proteobacteria
Spirochaetales
Nitrospirales
Alphaproteobacteria
Synergistales
Spirochaetes
Nitrospira
Synergistia
Deinococci
Nitrospirae
Chthoniobacteraceae
Chthoniobacterales
Thermi
Verrucomicrobia
Veillonellaceae
Clostridia
Bacteria
Firmicutes
Tissierellaceae
Actinobacteria
Ruminococcus
Ruminococcaceae
Oscillospira
Chloroflexi
Peptococcaceae
Clostridiales
Bacteroidetes
Desulfotomaculum
Actinobacteria
Actinomycetales
Lachnospiraceae
Bacilli
Microbacteriaceae
Lactobacillales
Bacteroidia
Cytophagia
Clostridiaceae
Streptococcaceae
Bacillales
Clostridium
Bacteroidales
Cytophagales
Streptomycetaceae
Paenibacillaceae
Bacillaceae
Bacteroidaceae
Porphyromonadaceae
Prevotellaceae
Streptomyces
Paenibacillus
Cytophagaceae
Chitinophagaceae
Flavobacteriaceae
Bacillus
Bacteroides
Prevotella
Flavobacterium
Nodes
0
1
278
378
1110
1510
2500
3400
4440
6040
6940
9440
10000
13600
(Percent Not Classified)^2
Number of Sequences

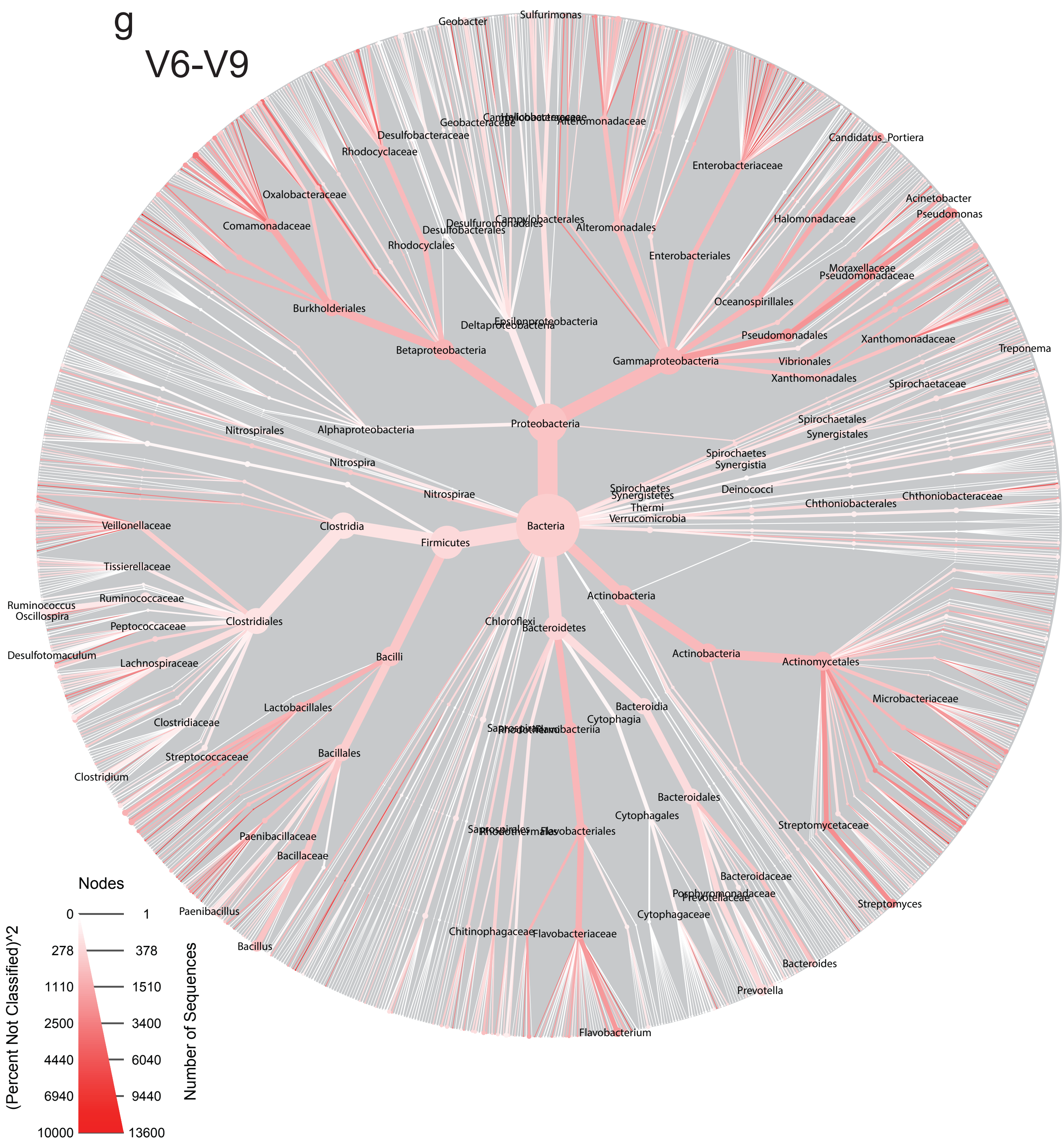

Supplementary Figure 2: Abundance and taxonomy of sequences used for *in silico* analysis. a) Phylogeny of all sequences used in 16S *in silico* analysis, in which node size and color reflects the number of sequences present in the original database at each taxonomic level. Subsequent panels show the same tree for b) V1-V2, c) V1-V3, d) V1-V9, e) V3-V5 f) V4, and g) V6-V9 sub-regions. In panels b-g node size reflects the number of sequences present in the original database and node colour reflects the percentage of sequences that could not be classified to species level using the respective sub-region. For clarity, labels are only shown for the top 100 most abundant taxa and the percent scale has been ^2 transformed. Source data are provided as a Source Data file.

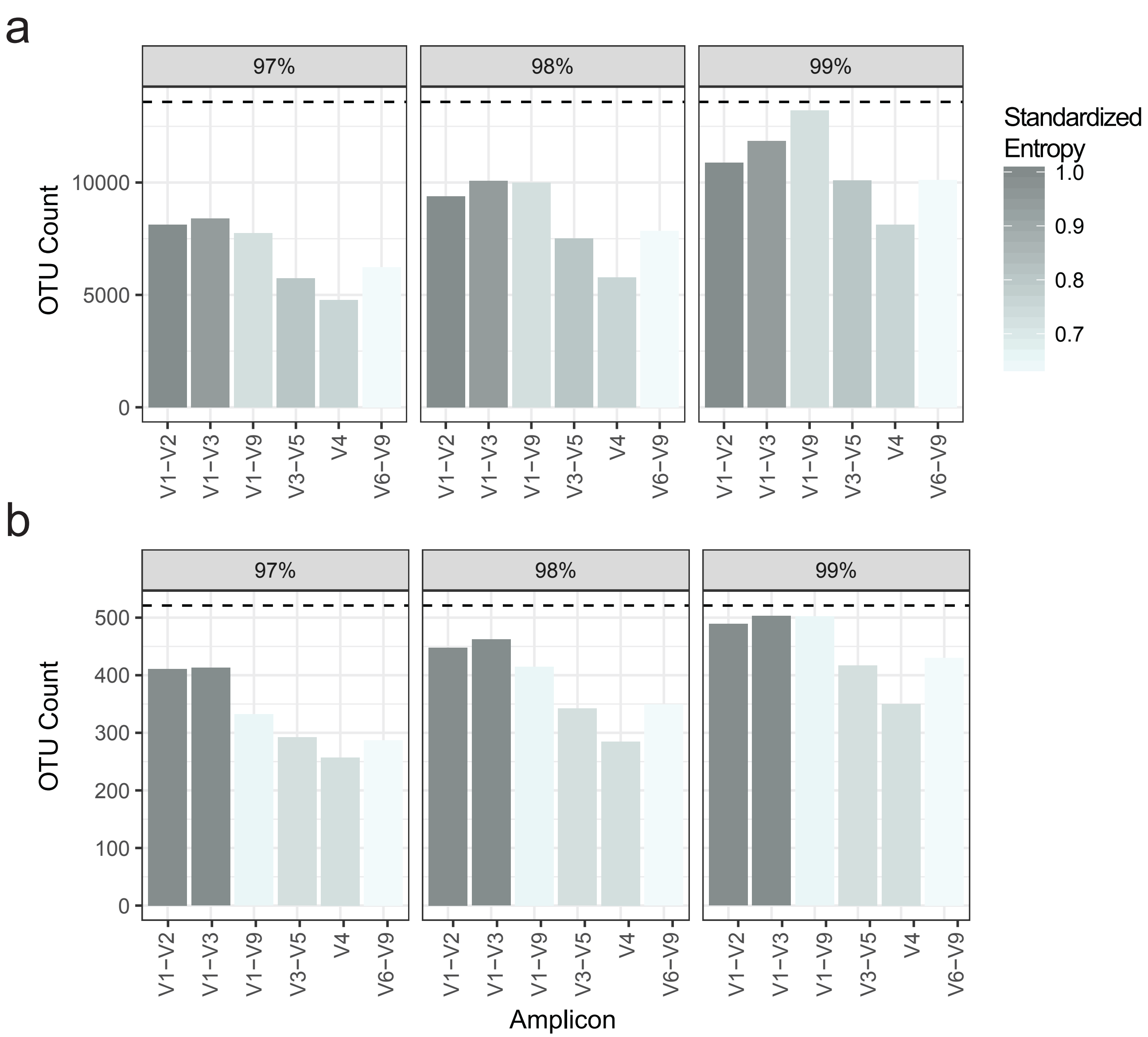

Supplementary Figure 3: The number of OTUs created when clustering *in silico* amplicons at different identity thresholds (97%, 98%, 99%) using USEARCH. Amplicons are generated from the a) Greengenes and b) HOMD databases. Dashed lines indicate the original number of unique sequences present in each database. Shading indicates the median per-base entropy across each variable region. Source data are provided as a Source Data file.

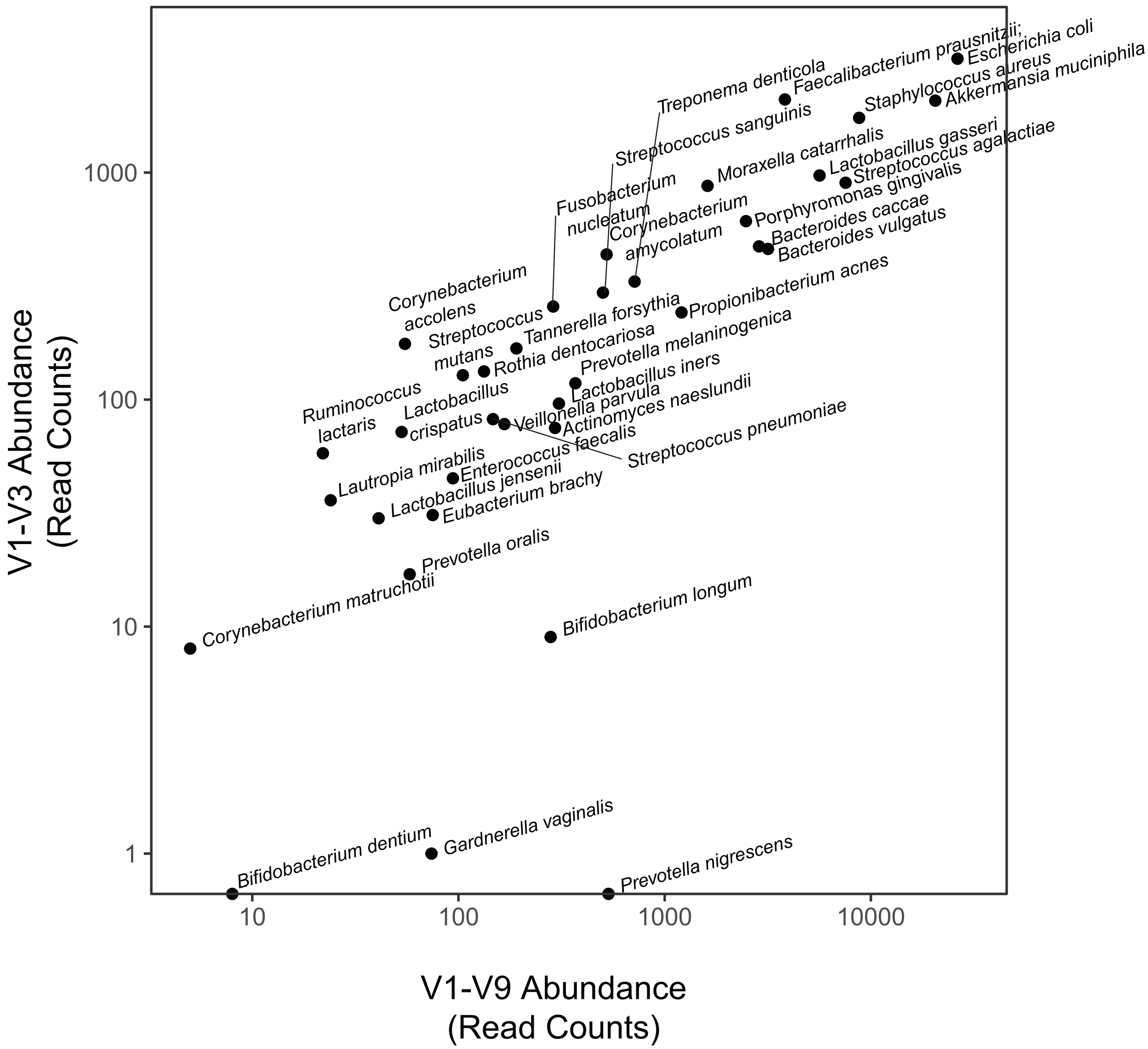

Supplementary Figure 4: Relative abundance of the 36 bacterial taxa present in the mock community, as estimated by sequencing of V1-V9 on the PacBio RS II platform (x axis) and sequencing of V1-V3 on the Illumina MiSeq platform (y axis). Source data are provided as a Source Data file.

a

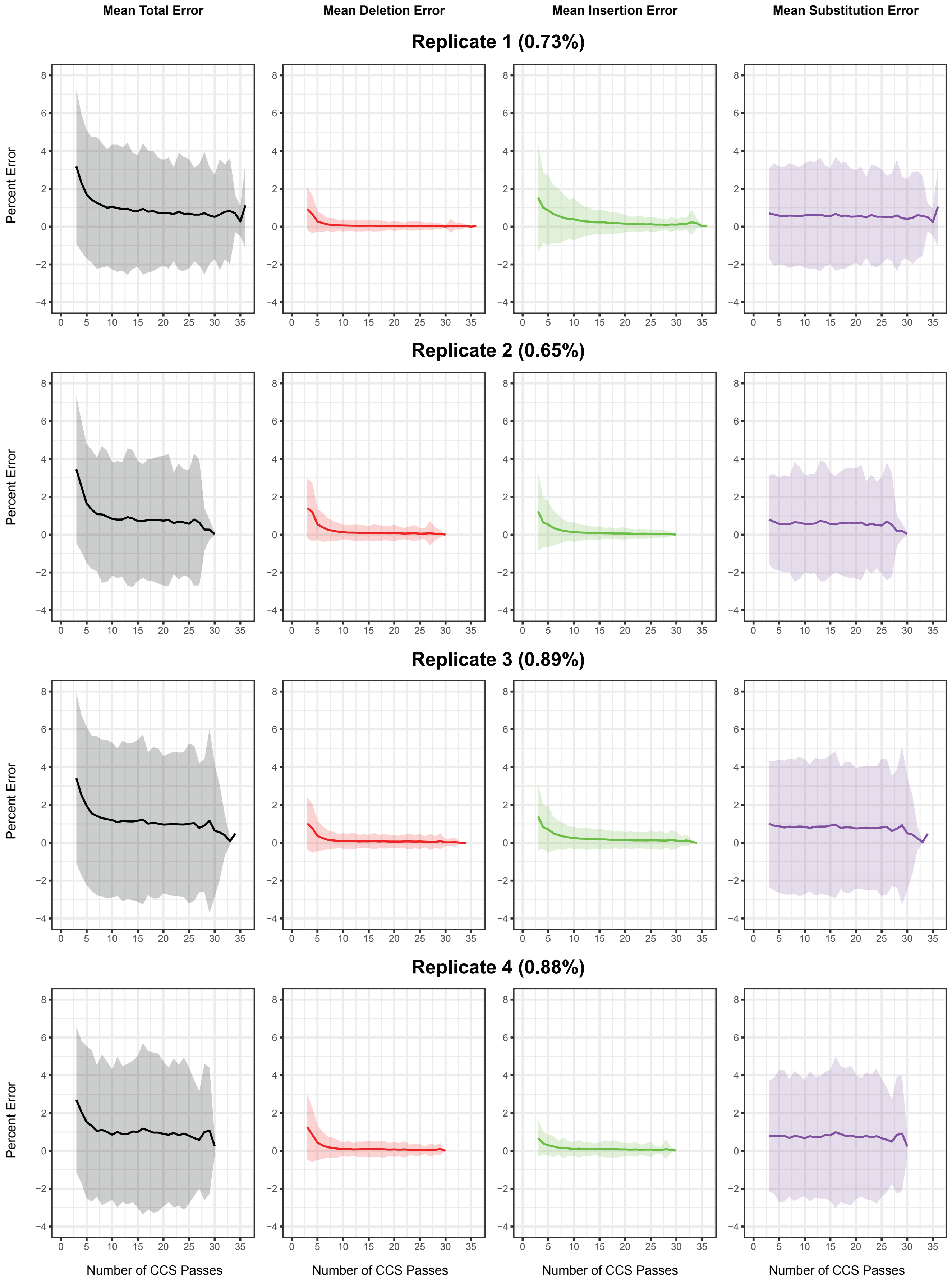

Mean Total Error
Mean Deletion Error
Mean Insertion Error
Mean Substitution Error
Replicate 1 (0.73%)
Replicate 2 (0.65%)
Replicate 3 (0.89%)
Replicate 4 (0.88%)
Percent Error
Number of CCS Passes

b

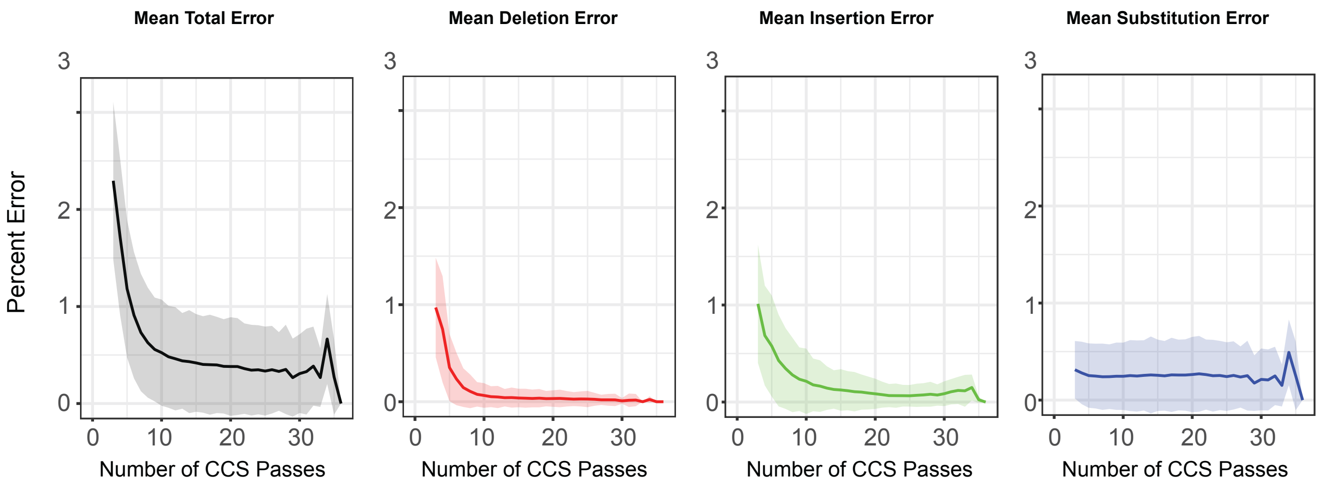

Supplementary Figure 5: Relationship between sequence alignment errors and number of passes used to generate PacBio CCS reads. Sequencing was performed on a PacBio RSII using P6C4v2 chemistry and CCS reads were processed using CCS2 v3.0.1. Errors were calculated by aligning V1-V9 amplicon sequences from the mock community to a reference database containing a single representative 16S sequence for each taxon. Shaded regions represent 2 standard deviations from the mean. Panel a) shows individual plots for four replicates of the mock community (numbers in parentheses indicate estimates of the minimum achievable total error). Replicate 1: n=30245, Replicate 2: n=19310, Replicate 3: n=24773, Replicate 4: n=14813. Panel b) shows summary plots for the combined sequence data from all four replicate runs. Source data are provided as a Source Data file.

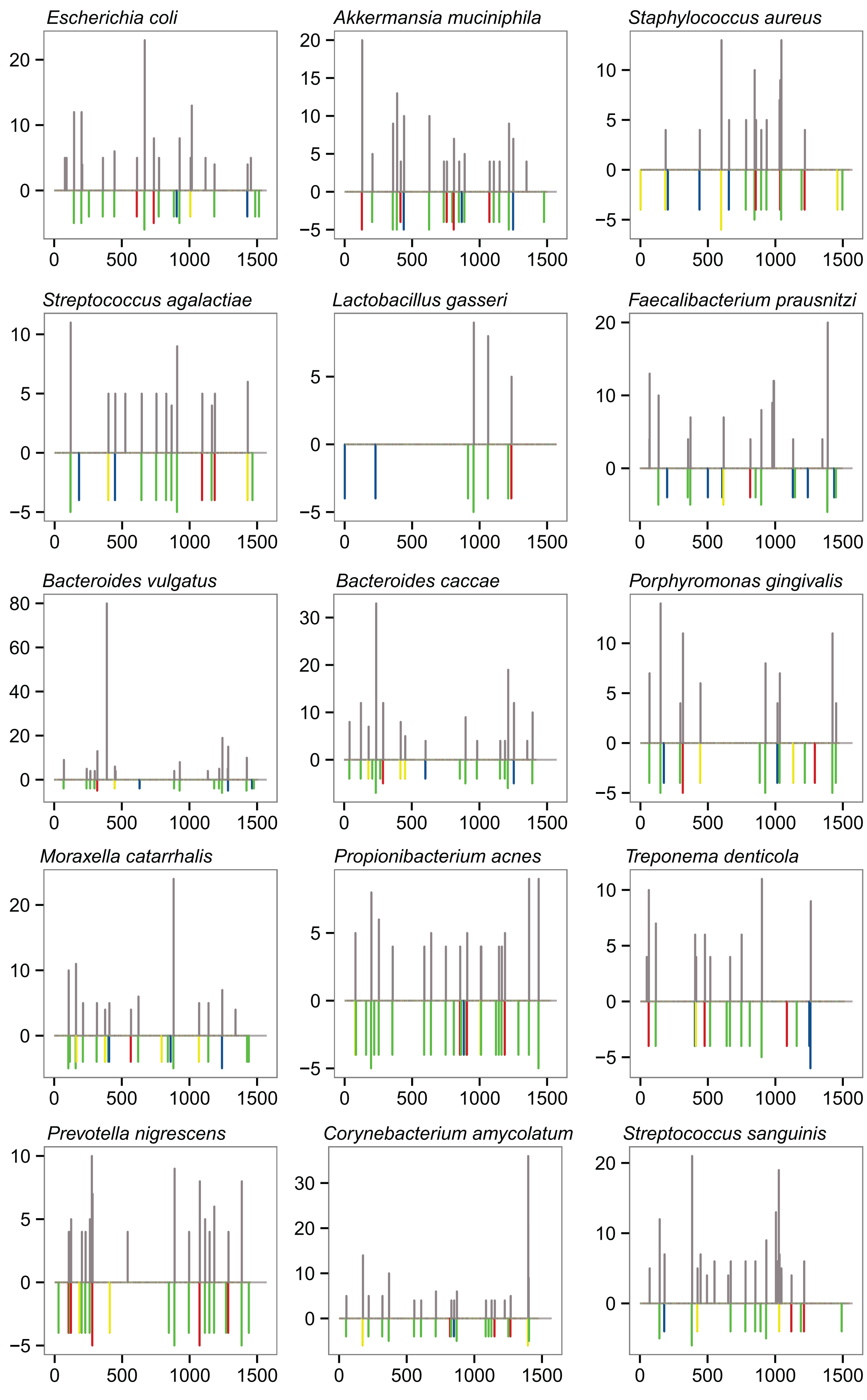
Escherichia coli
Akkermansia muciniphila
Staphylococcus aureus
Streptococcus agalactiae
Lactobacillus gasseri
Faecalibacterium prausnitzi
Bacteroides vulgatus
Bacteroides caccae
Porphyromonas gingivalis
Moraxella catarrhalis
Propionibacterium acnes
Treponema denticola
Prevotella nigrescens
Corynebacterium amycolatum
Streptococcus sanguinis
0
500
1000
1500

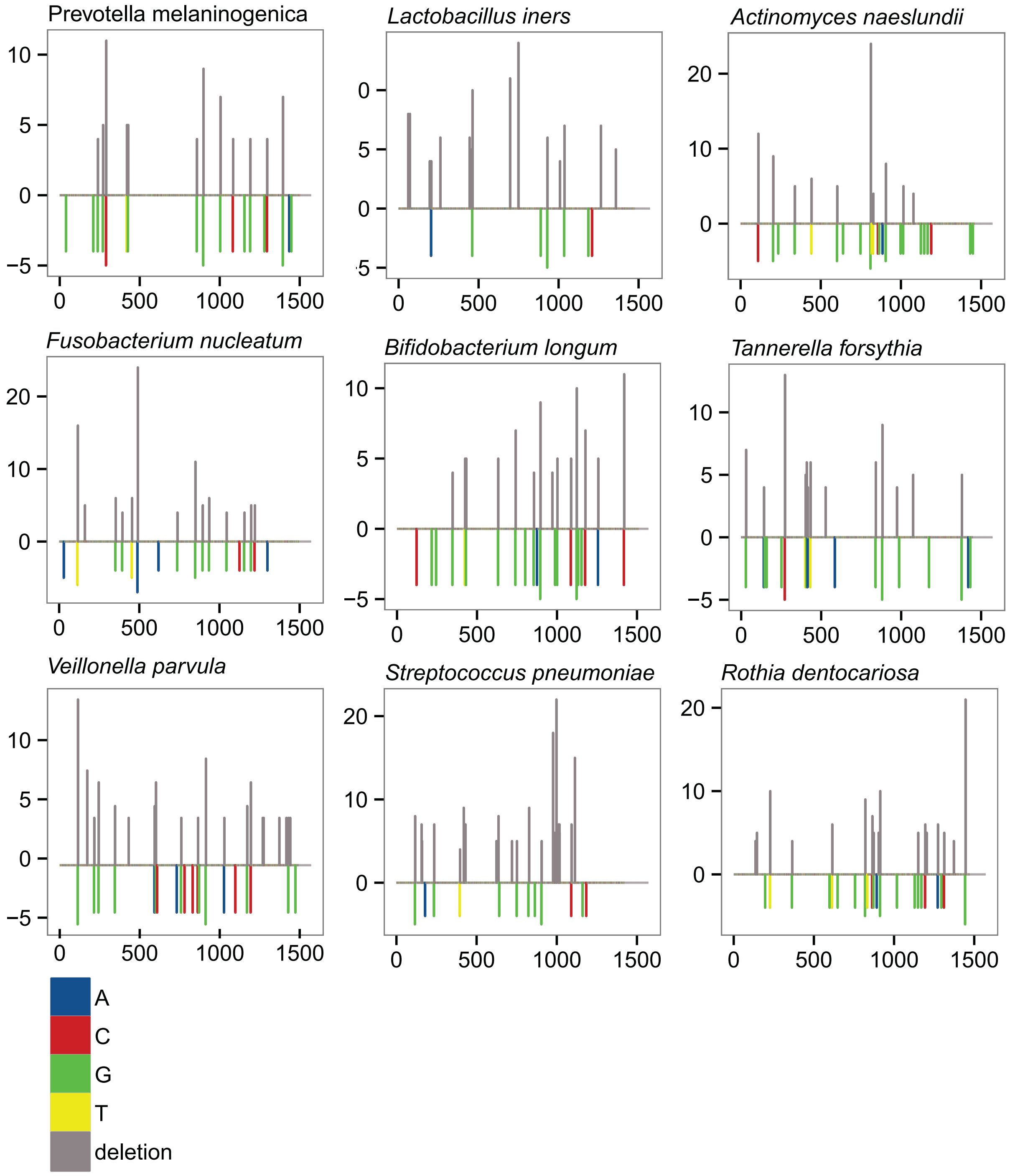

Supplementary Figure 6: Coincidence of deletion errors in PacBio CCS alignments with homopolymer runs. For each plot, the upper panel depicts the percent deletions occurring within aligned sequences at each based position along the reference 16S rRNA gene sequence. The lower inverted panel depicts the location and size of homopolymers within the reference sequence (only homopolymers greater than four bases in length are shown). Plots are shown for mock community taxa with more than100 aligned CCS reads (Supplementary Figure 4). Source data are provided as a Source Data file.

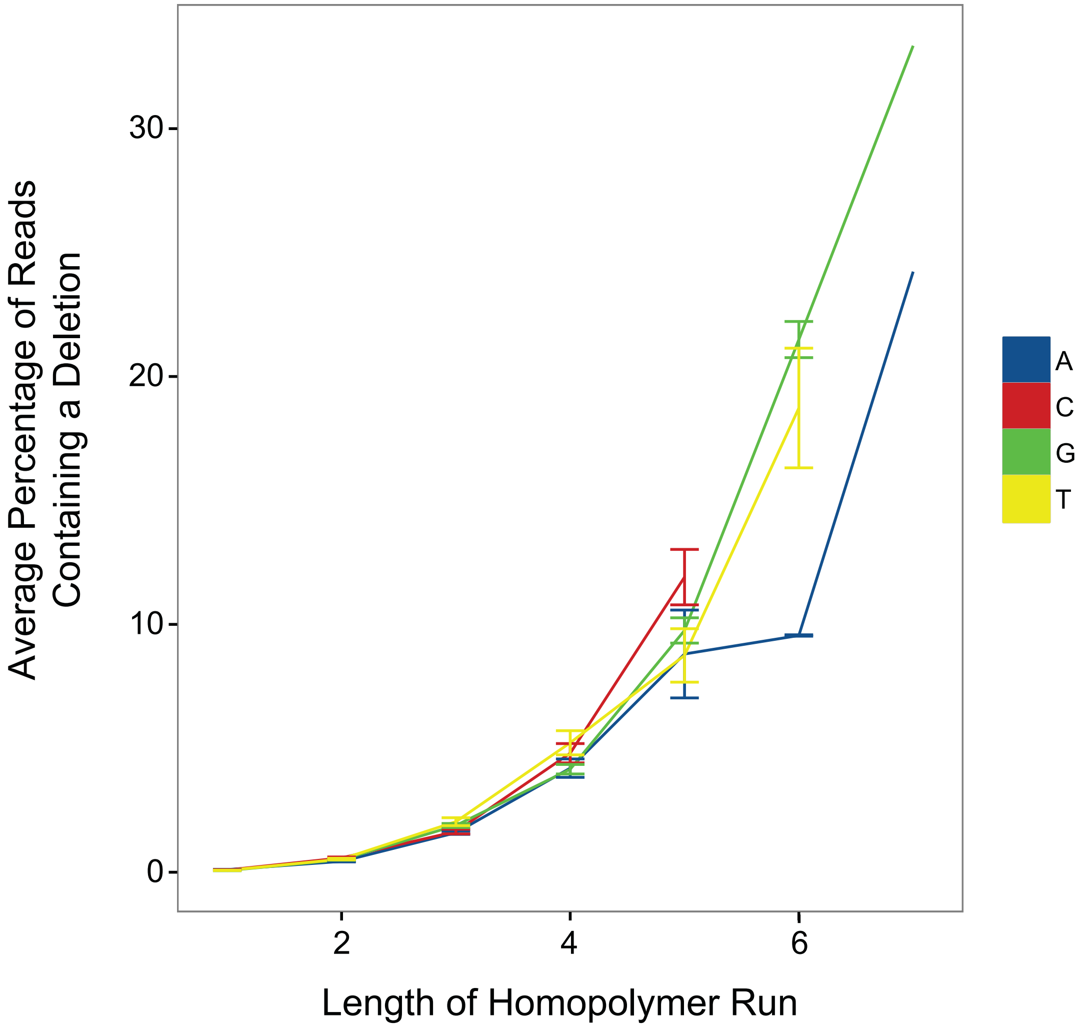

Length of Homopolymer Run	Number of Occurences			
	A	C	G	T
1	6560	7177	7880	6963
2	2312	1638	2634	1607
3	595	441	705	262
4	64	77	299	58
5	11	14	80	6
6	2	0	7	9
7	1	0	1	0

Length of Homopolymer Run	Number of Reads			
	A	C	G	T
1	16034166	17441613	19332347	16750088
2	5934672	4084385	6316552	3795109
3	1524410	1013143	1553297	610713
4	168075	153837	616268	95445
5	48080	76051	239618	4096
6	710	0	35799	9807
7	260	0	2714	0

Supplementary Figure 7: Relationship between frequency of deletion errors in PacBio CCS alignments and the length of coincident homopolymer runs in the reference sequence. Sequence data were generated from the microbial mock community and aligned to a database containing single representative 16S rRNA gene sequence for each taxon. Lower left table shows the number of homopolymer runs present in reference sequences. Lower right table shows the number of reads covering each type of homopolymer run. Source data are provided as a Source Data file.

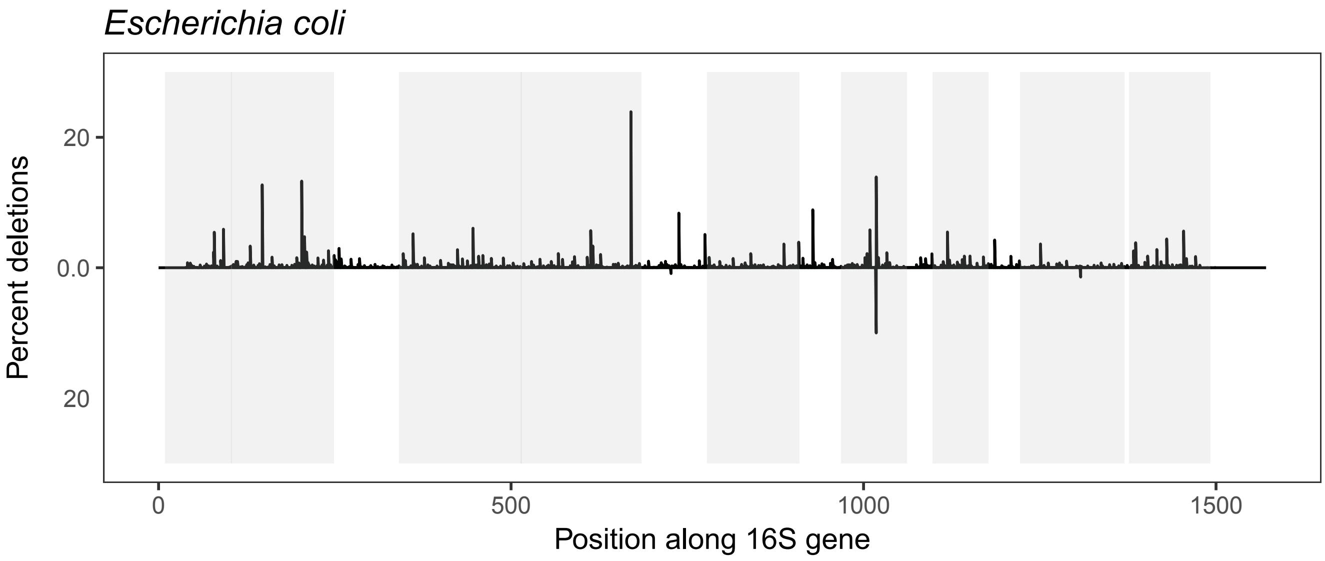

Supplementary Figure 8: Location of deletion errors observed in PacBio CCS reads (upper panel) and Illumina MiSeq reads (lower, invert- ed panel) aligned to a single *E. coli* 16S rRNA gene reference sequence. Sequence data were generated from the bacterial mock community. Source data are provided as a Source Data file.

Replicate 1 (8764 sequences aligned)

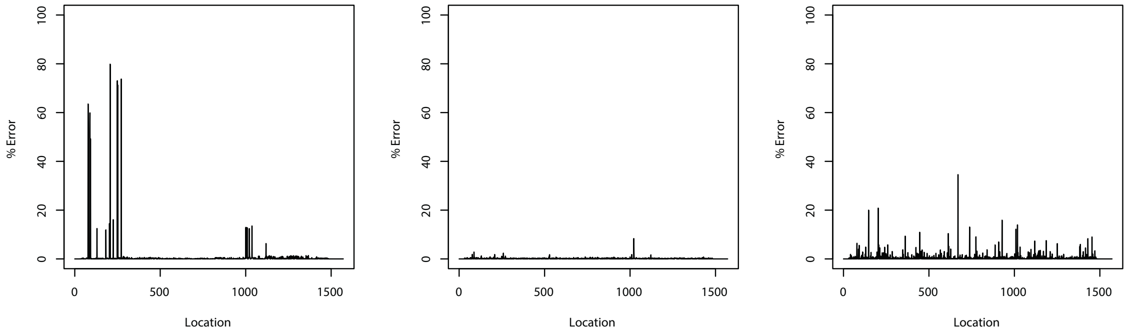

Replicate 2 (5721 sequences aligned)

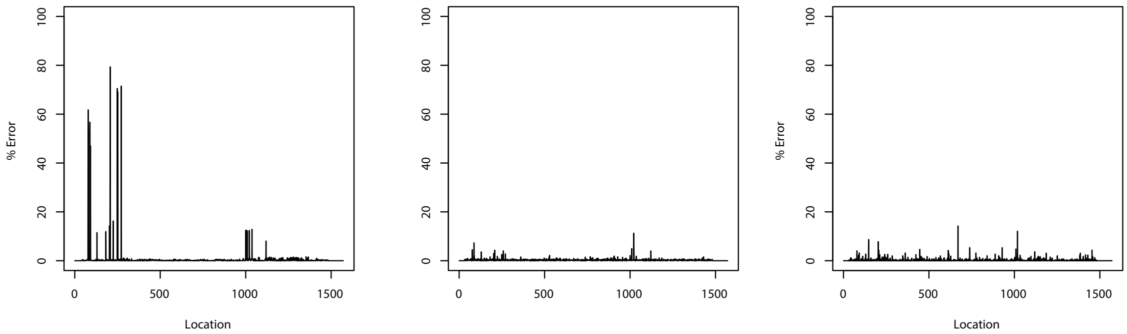

Replicate 3 (7362 sequences aligned)

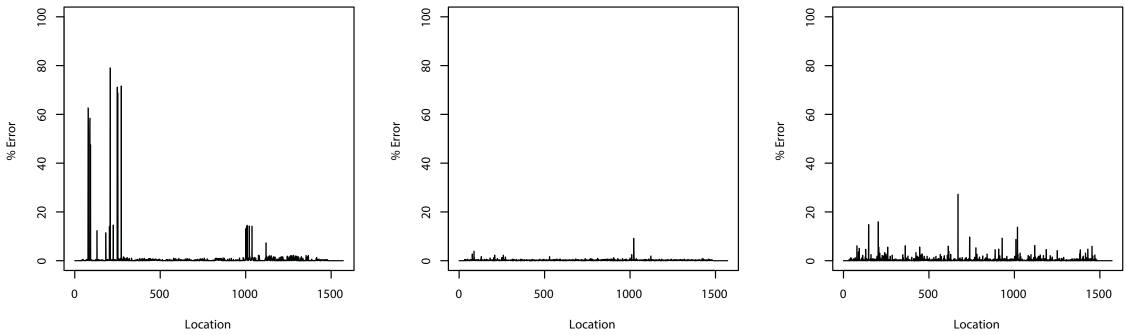

Replicate 4 (4477 sequences aligned)

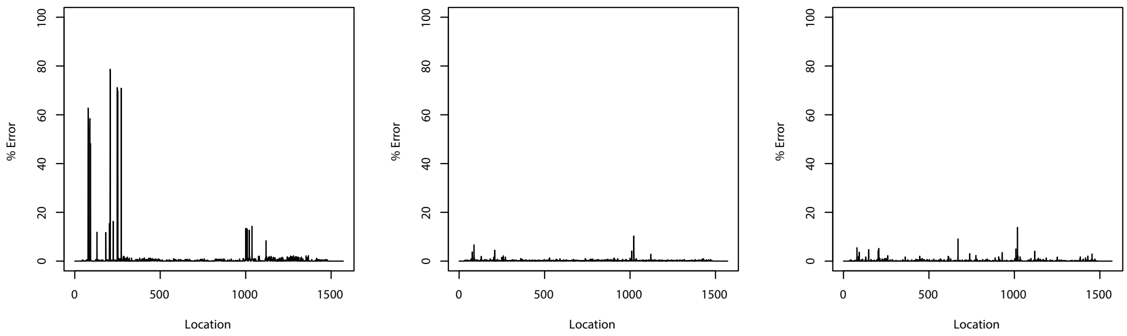

Supplementary Figure 9: Substitution, insertion, and deletion errors in sequences aligned to a single reference 16S gene sequence for E. coli str. K-12 MG1655. Plots are shown separately for each of the four replicate samples of the 36 species mock community. Source data are provided as Source Data file.

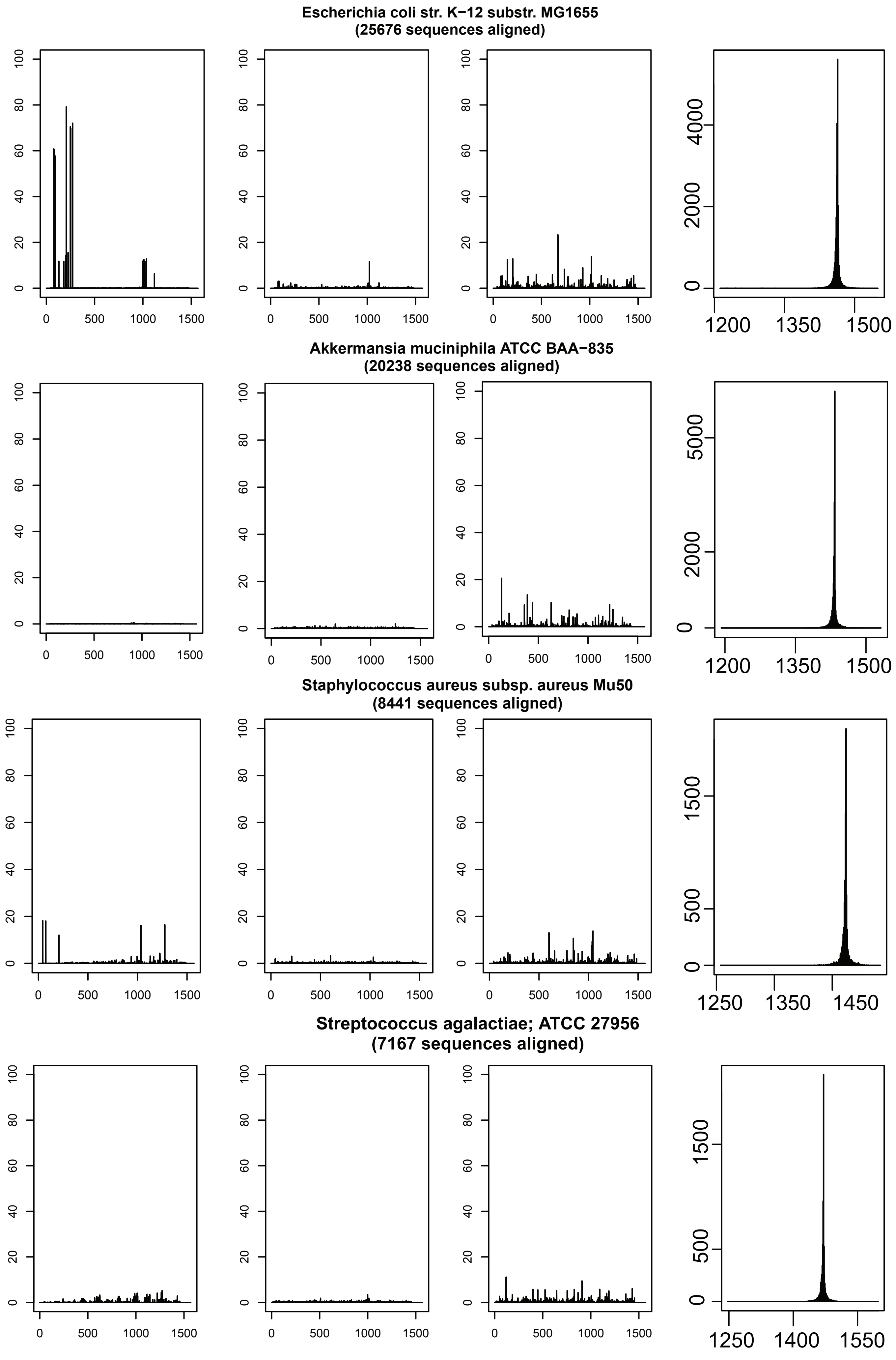
Escherichia coli str. K−12 substr. MG1655
(25676 sequences aligned)
Akkermansia muciniphila ATCC BAA−835
(20238 sequences aligned)
Staphylococcus aureus subsp. aureus Mu50
(8441 sequences aligned)
Streptococcus agalactiae; ATCC 27956
(7167 sequences aligned)

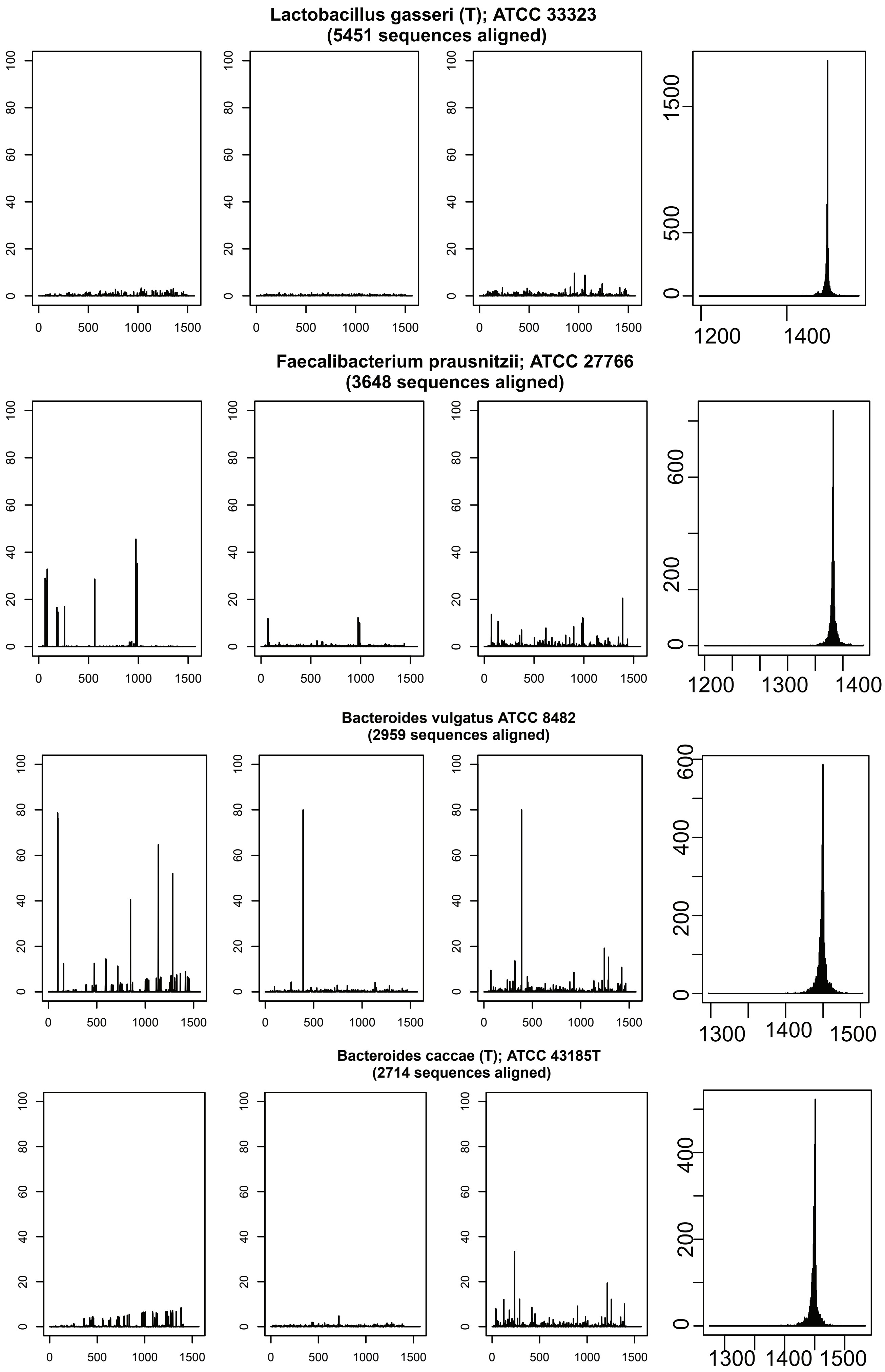

Lactobacillus gasseri (T); ATCC 33323
(5451 sequences aligned)
Faecalibacterium prausnitzii; ATCC 27766
(3648 sequences aligned)
Bacteroides vulgatus ATCC 8482
(2959 sequences aligned)
Bacteroides caccae (T); ATCC 43185T
(2714 sequences aligned)

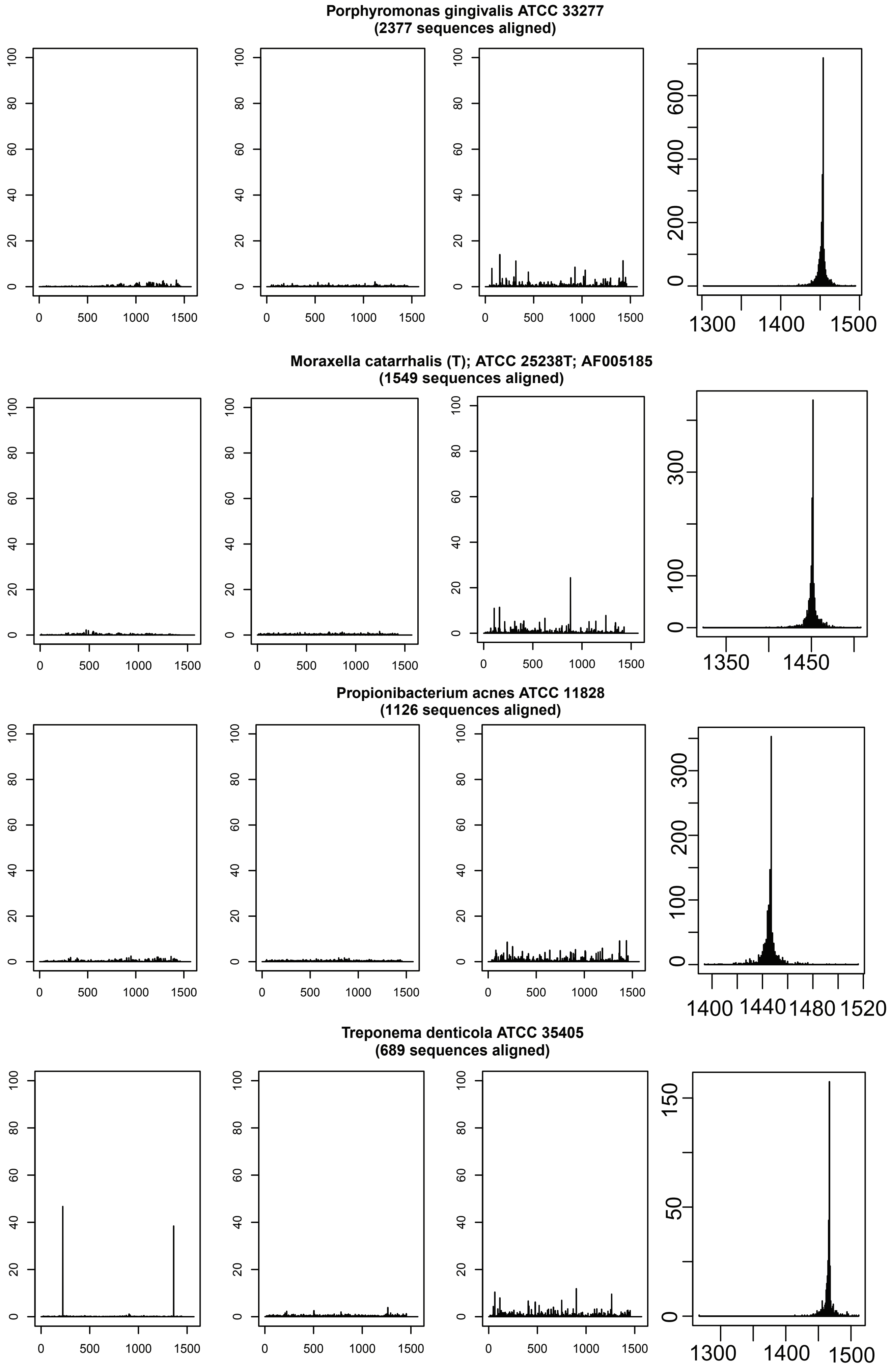
Porphyromonas gingivalis ATCC 33277
(2377 sequences aligned)
Moraxella catarrhalis (T); ATCC 25238T; AF005185
(1549 sequences aligned)
Propionibacterium acnes ATCC 11828
(1126 sequences aligned)
Treponema denticola ATCC 35405
(689 sequences aligned)

Prevotella nigrescens (T); NCTC 9336; X73963
(487 sequences aligned)

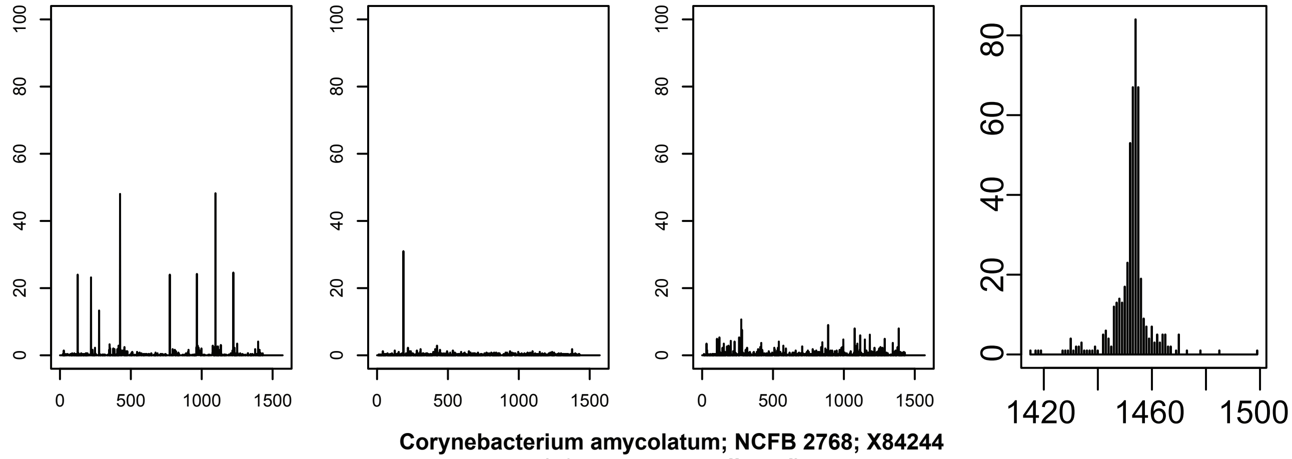

Corynebacterium amycolatum; NCFB 2768; X84244
(465 sequences aligned)

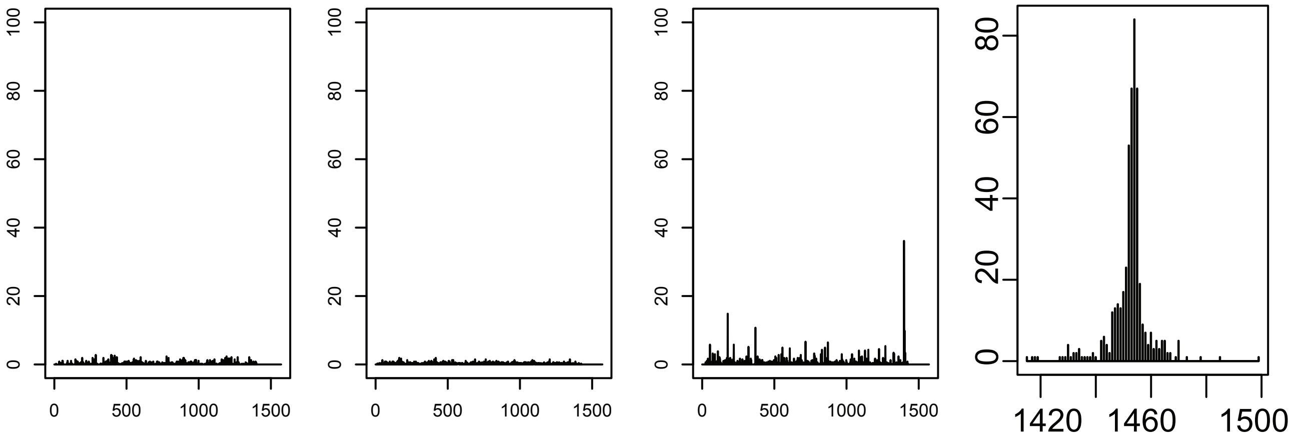

Streptococcus sanguinis SK36
(430 sequences aligned)

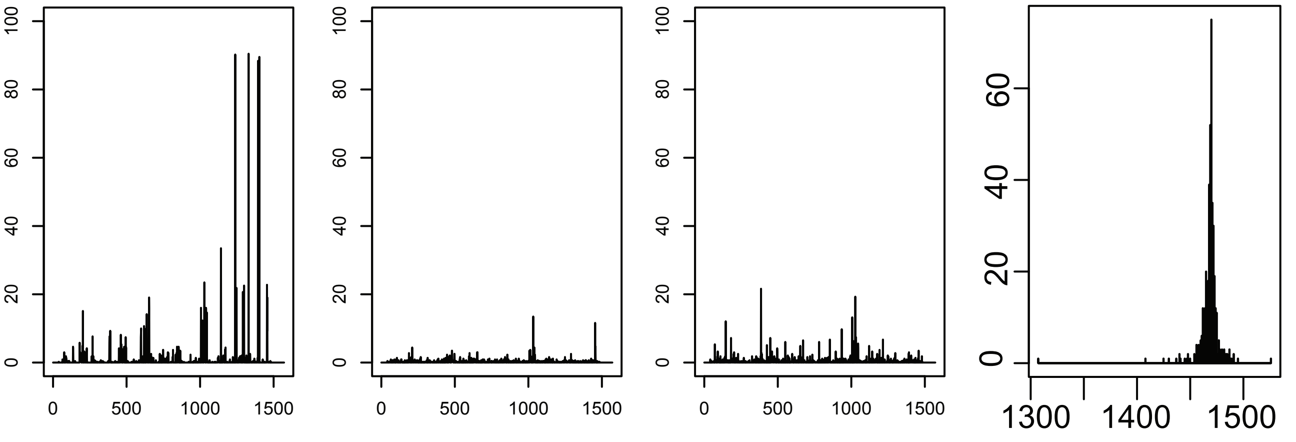

Prevotella melaninogenica; ATCC 25845
(327 sequences aligned)

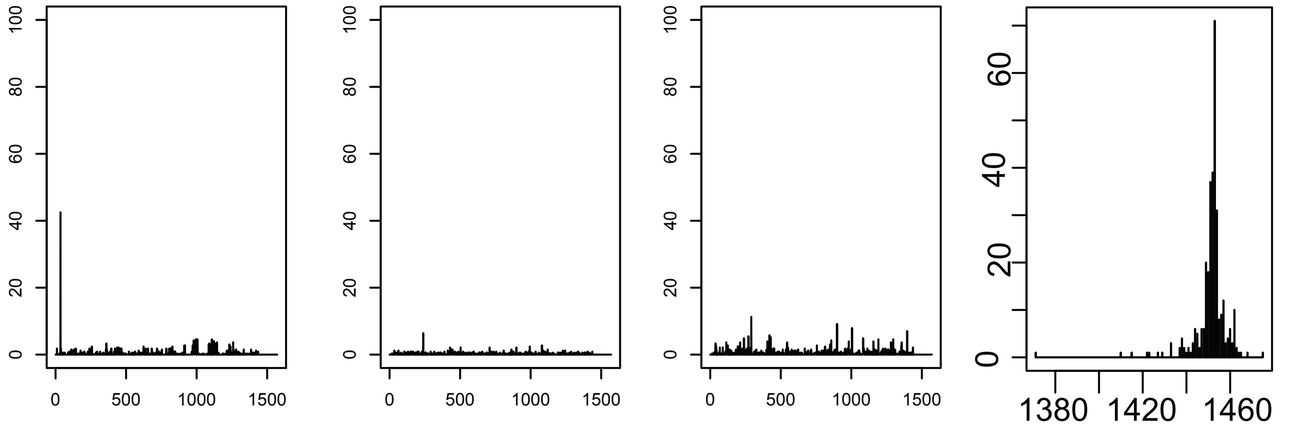

Lactobacillus iners; VA15_2004; AY526083
(292 sequences aligned)

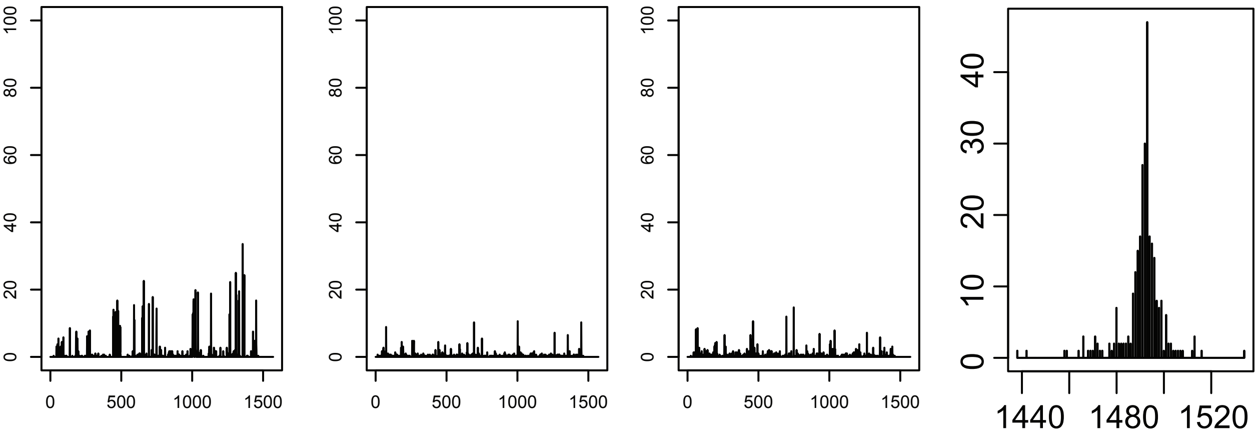

Actinomyces naeslundii; CCUG 33519
(262 sequences aligned)

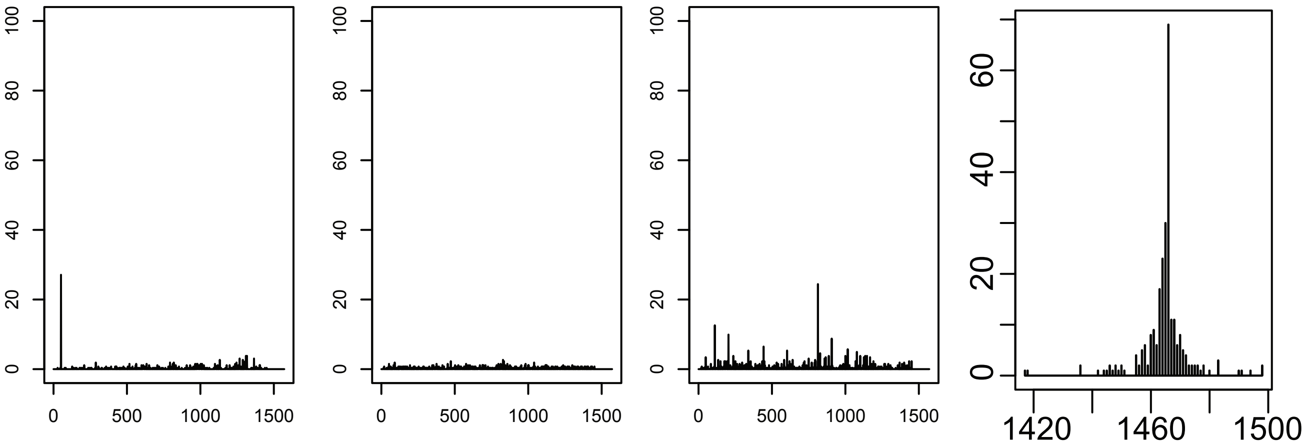

Fusobacterium nucleatum (T); ATCC 25586
(260 sequences aligned)

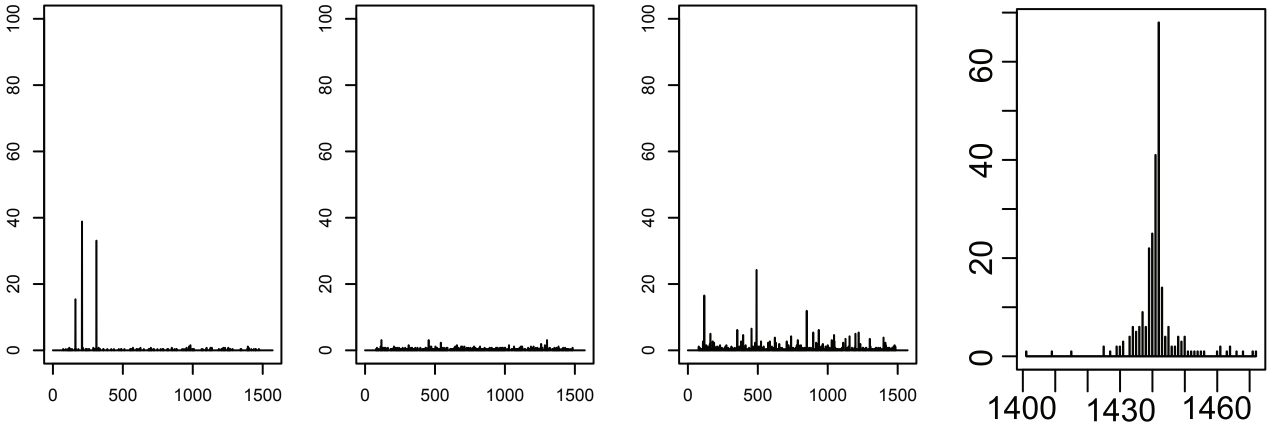

Bifidobacterium longum (T); ATCC 15697
(252 sequences aligned)

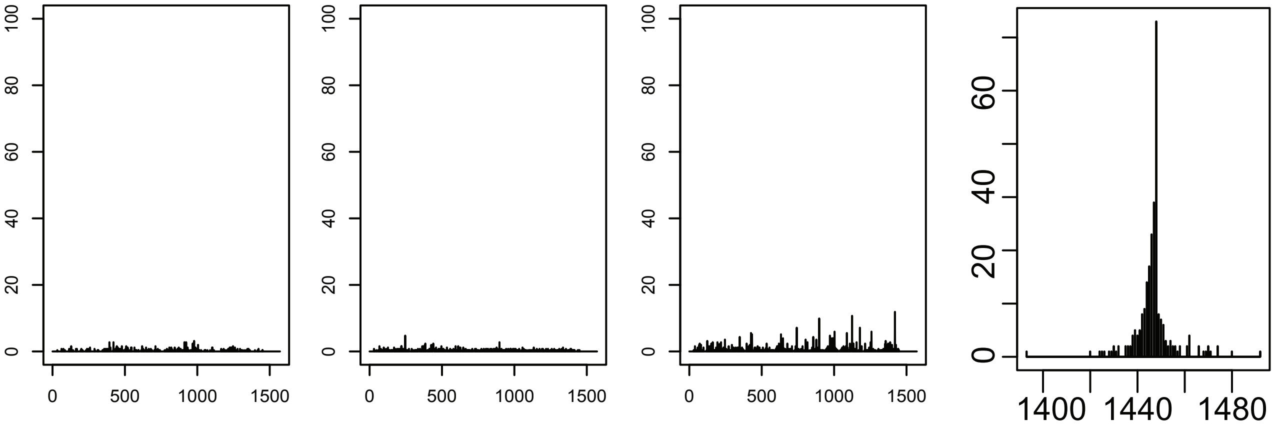

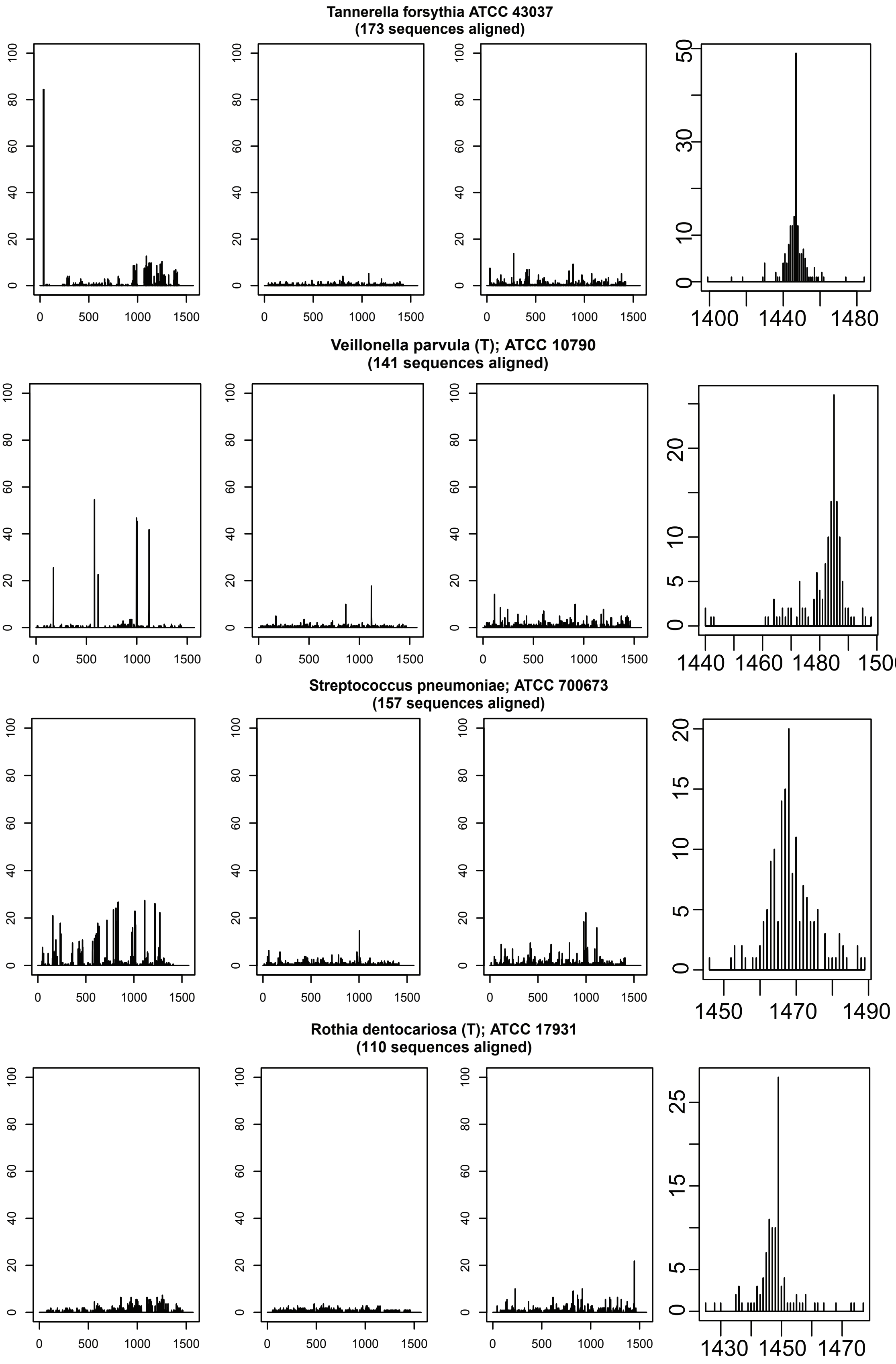

Supplementary Figure 10 Location and frequency of alignment errors in PacBio CCS reads aligned to 16S rRNA reference gene sequences. Panels are as follows far left: substitution errors, mid-left: insertion errors, mid-right: deletion errors, far right: histogram showing distribution in length of successfully aligned regions. Sequence data were generated from the 36 taxon bacterial mock community; however, panels are only shown for taxa with >100 aligned CCS reads.

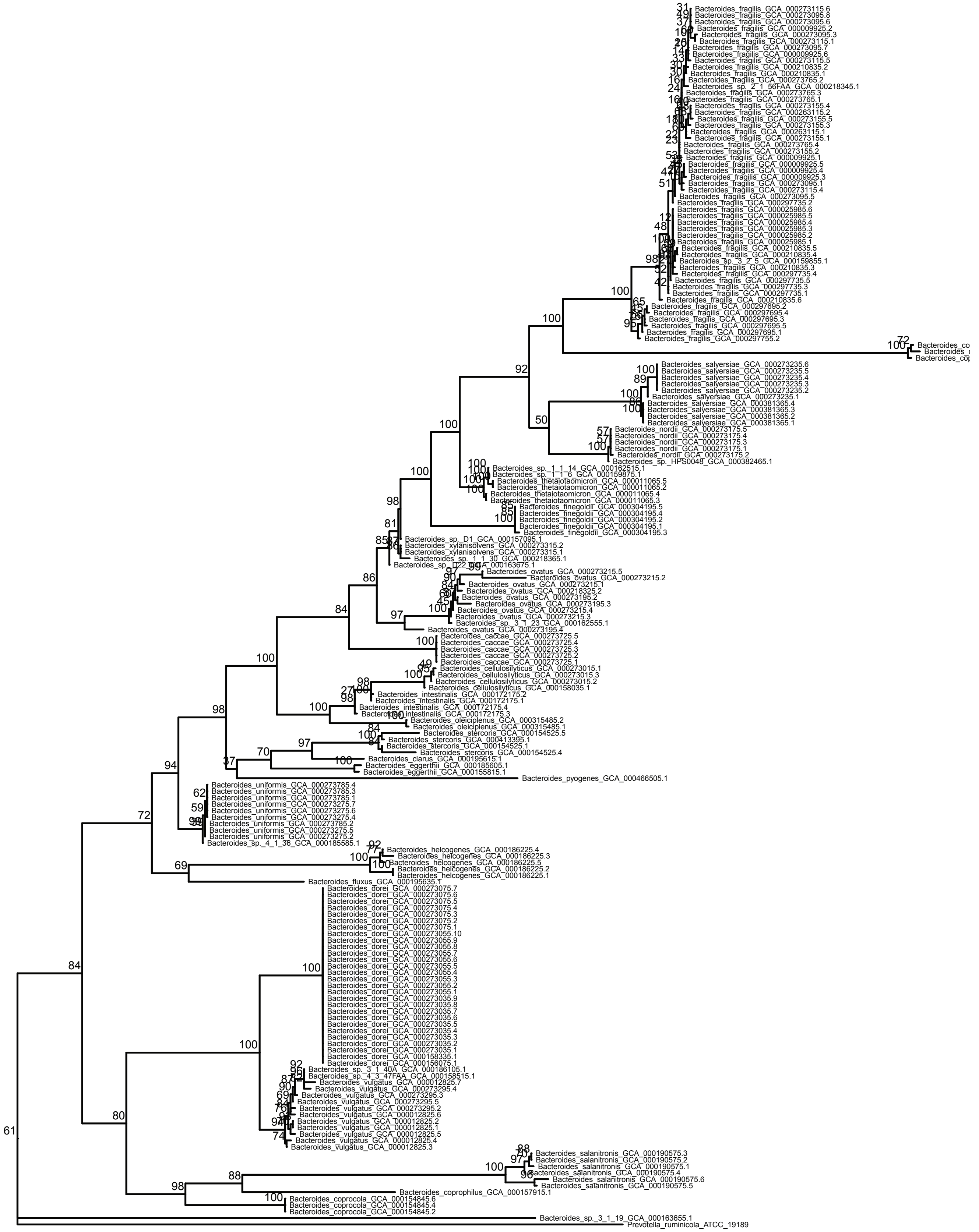

Supplementary Figure 11: Maximum likelihood tree showing the inferred phylogenetic relationship between all *Bacteroides* 16S rRNA gene sequences present in the Real Time Genomics (RTG) reference database. Bootstrap confidence estimates are provided for each tree branch.

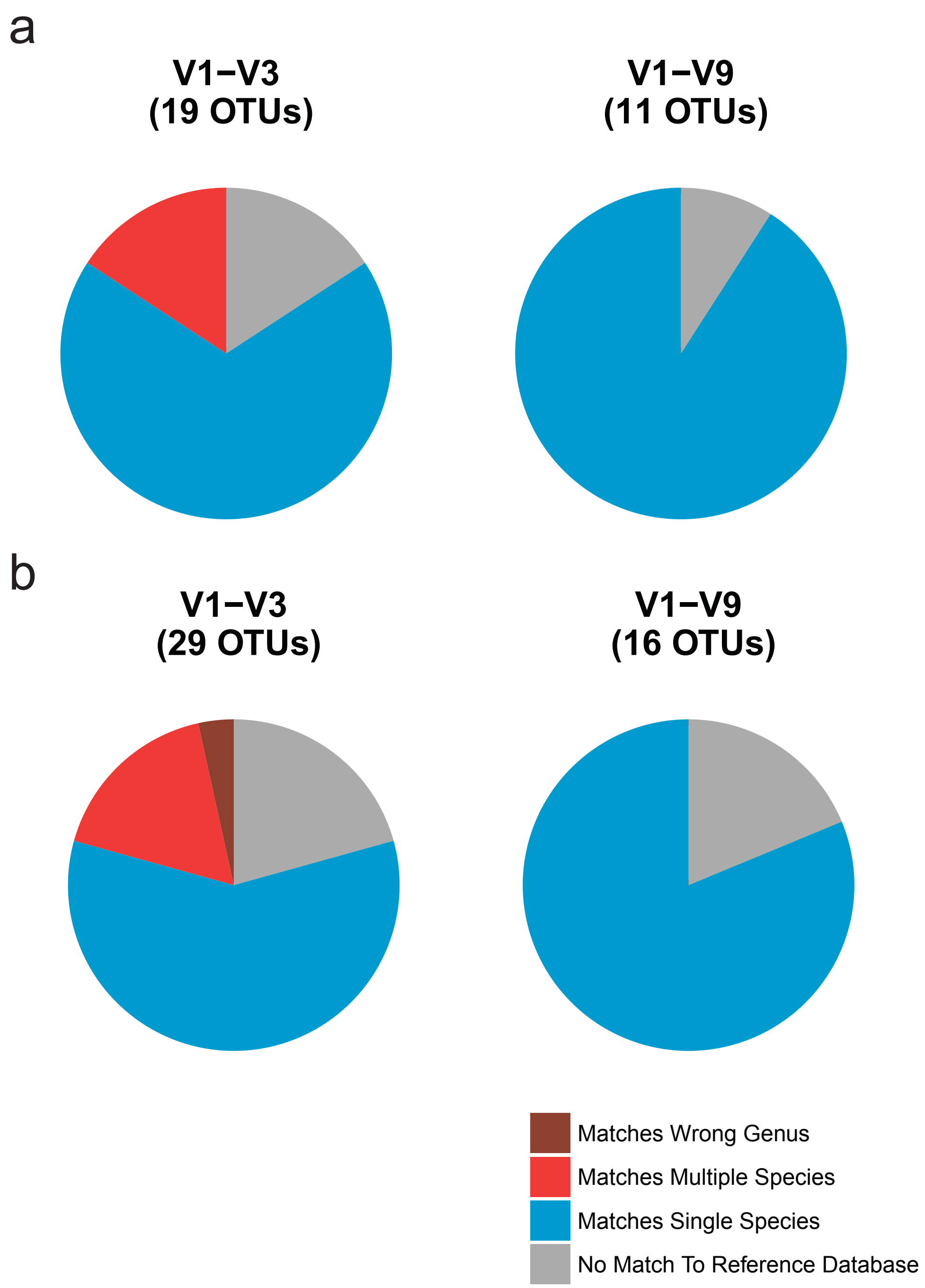

Supplementary Figure 12: The proportion of *Bacteroides* OTUs that could be assigned to a species by aligning the OTU sequence to the RTG reference database. Results are shown for V1-V3 amplicons generated using the Illumina MiSeq platform and V1-V9 amplicons generated using the PacBio RS II platform. OTU clustering was performed at a) 97% and b) 99% similarity thresholds.

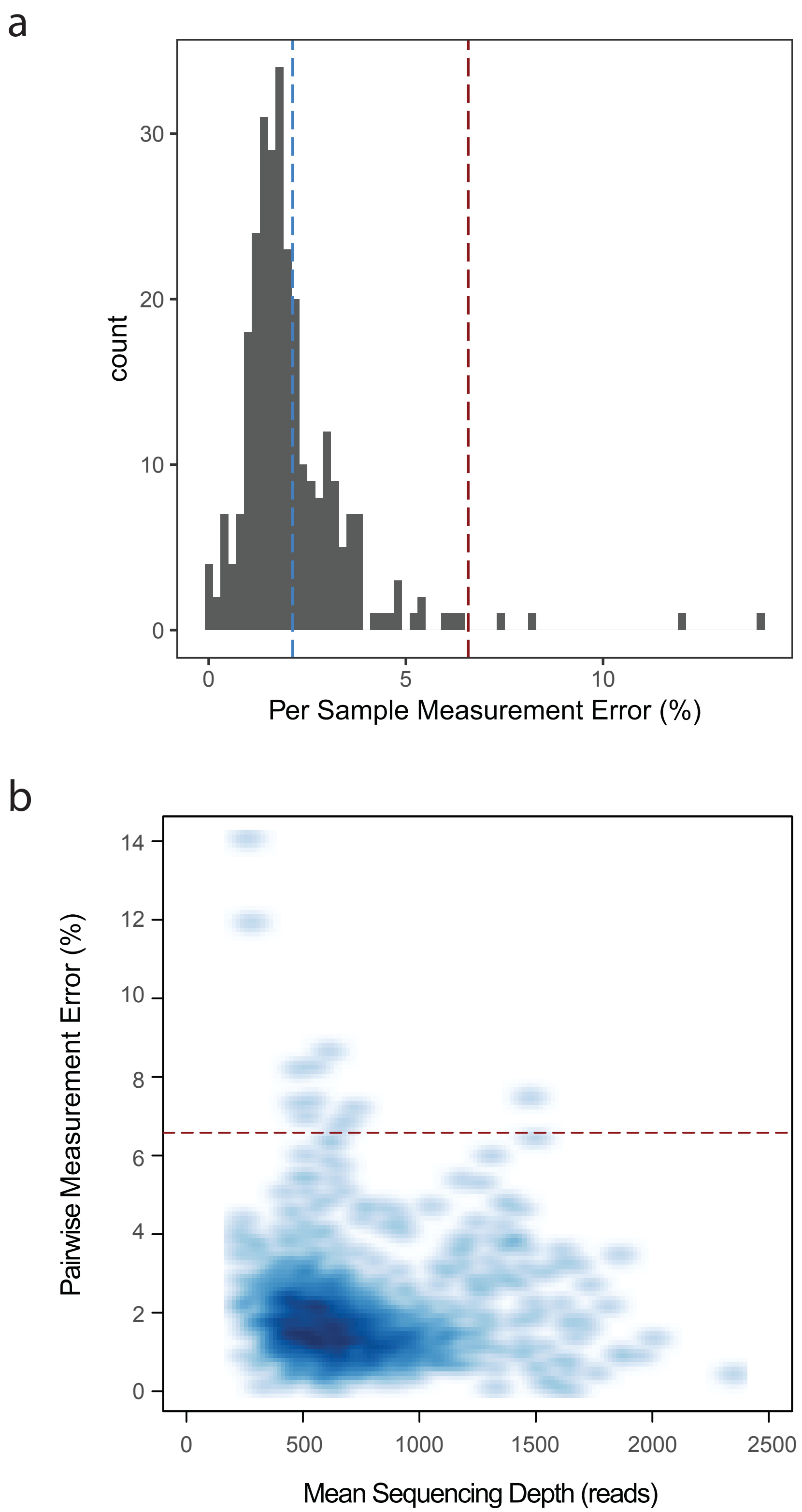

Supplementary Figure 13: Estimating measurement error attributable to sequencing platform and depth. a) Histogram showing the distribution in measurement error calculated for samples that were sequenced two or more times at different loading concentrations. b) Relationship between measurement error and mean sequencing depth for pairwise comparisons of the sequencing replicates for each sample. Dashed lines indicate the mean measurement error (blue) and the upper boundary for expected measurement error (red, calculated as 3 standard deviations above the mean measurement error) samples with measurement error above this upper boundary were dropped from subsequent analyses. Source data are provided as a Source Data file.

Supplementary Table 1: Primers used to annotate variable regions within 16S rRNA gene sequences.

16S Subregion	Forward Name	Forward Sequence	Reverse Name	Reverse Sequence	Expected size (bases)
V1-V2	27F	AGAGTTTGATCMTGGCTCAG	337R	CYIACTGCTGCCTCCCGTAG	310
V4	515F	GTGCCAGCMGCCGCGGTAA	806R	GGACTACHVGGGTWTCTAAT	219
V1-V3	27F	AGAGTTTGATCCTGGCTCAG	534R	ATTACCGCGGCTGCTGG	507
V3-V5	357F	CCTACGGGAGGCAGCAG	926R	CCGTCAATTCMTTTRAGT	569
V6-V9	968F	AACGCGAAGAACCTTAC	1492R	TACGGYTACCTTGTTAYGACTT	524
V1-V9	27F	AGAGTTTGATCMTGGCTCAG	1492R	TACGGYTACCTTGTTAYGACTT	1465

Supplementary Table 2: Summary of sequences curated from public databases for *in silico* analysis. a) Summary of the number of sequences remaining after curation. b) Summary of sequences discarded from greengenes database at each curation step. c) Summary of sequences discarded from HOMD at each curation step. During curation sequences were discarded if a match could not made be for one or more of the primers that delineate the variable regions included in this study, if a sequence contained ambiguous bases, or if the *in silico* sequence generated for a variable region was an unusual length (see Online Methods).

a

Database	Number of Sequences in Database With Genus-Level Identification	Number of Sequences Remaining After Filtering	Percent Sequences Remaining After Filtering
greengenes	93,463	13,587	15%
homd	832	521	75%

b

Reason for discarding	Number of Sequences Discarded Per Subregion					
	V1-V2	V1-V3	V1-V9	V3-V5	V4	V6-V9
No primer match	3630	1374	47540	5	13	2656
No forward primer match	53322	53502	10383	2301	1494	1753
No reverse primer match	905	346	18415	180	326	59161
Amplicon too short	1851	3369	153	340	827	192
Amplicon contains Ns	719	992	1055	5959	3826	1075

c

Reason for discarding	Number of Sequences Discarded Per Subregion					
	V1-V2	V1-V3	V1-V9	V3-V5	V4	V6-V9
No primer match	0	0	0	0	0	5
No forward primer match	16	1	16	1	1	15
No reverse primer match	0	2	76	0	0	65
Amplicon too short	24	24	27	11	17	24
Amplicon contains Ns	14	19	45	21	11	23

Supplementary Table 3: Details of the 36 taxa present in the bacterial mock community. (a) 16 rRNA copy number was not available, thus it was calculated from the average copy number of the available species belonging to the same genus. (b) 16 rRNA copy number was not available neither in species nor the same genus, thus it was calculated from the average of the copy number of the species included in this mock community. (c) 16 rRNA copy number was not available thus it was calculated from the average copy number of the available strains belonging to the same species. (d) Genomic DNA was purchased directly from ATCC. (e) Supplemented with the same concentrations of cysteine and haemin and 0.6% (volume/volume) of lactic acid. (f) Supplemented with 0.5 g liter−1 cysteine. (g) Supplemented with 0.5 g liter−1 cysteine, 5 mg liter−1 hemin and 1 mg liter−1 menadione. Acronyms: BHI – Brain-Heart Infusion, TSB – Tryptic soy broth.

36 bacterial mock species	Body site	16S rRNA copies per genome	Strain (ATCC number)	Bacterial Culture method
Corynebacterium accolens	airway	4.3 (a)	49725	ATCC medium 44, 260
Moraxella catarrhalis	airway	4.5 (b)	25240D-5 (d)	
Streptococcus pneumoniae	airway	4.0	700673	ATCC medium 44, 260
Akkermansia muciniphila	gut	3.0	BAA-835D-5 (d)	
Bacteroides caccae	gut	6.0 (a)	43185	ATCC medium 1490
Bacteroides vulgatus	gut	7.0	8482D-5 (d)	
Enterococcus faecalis	gut	4.0 (c)	47077	ATCC medium 2836
Escherichia coli	gut	7.0 (c)	700926D-5 (d)	
Faecalibacterium prausnitzii	gut	4.5 (b)	27766	ATCC medium 1703
Ruminococcus lactaris	gut	4.5 (b)	29176	ATCC medium 1490, 260
Bifidobacterium dentium	gut/oral cavity	4.0 (c)	27678	ATCC medium 2107, 260
Bifidobacterium longum subsp. *infantis*	gut/oral cavity	3.7 (c)	15697D-5 (d)	
Prevotella oralis	gut/oral cavity	4.0 (a)	33269	ATCC medium 1490
Actinomyces naeslundii MG-1	oral cavity	3.0	43146 (d)	BHI medium
Corynebacterium matruchotti	oral cavity	4.3 (a)	33806	ATCC medium 1490
Eubacterium brachy	oral cavity	5.0 (a)	33089	ATCC medium 1015
Fusobacterium nucleatum subsp. *nucleatum*	oral cavity	5.0	25586	BHI medium (f)
Lautropia mirabilis	oral cavity	4.5 (b)	51599	ATCC medium 814
Porphyromonas gingivalis	oral cavity	4.0	33277	TSB (g)
Prevotella melaninogenica	oral cavity	4.0 (a)	25845	ATCC medium 2863
Prevotella nigrescens	oral cavity	4.0 (a)	33563	ATCC medium 2722, 260
Rothia dentocariosa	oral cavity	3.0 (a)	17931	ATCC medium 44
Streptococcus mutans	oral cavity	5.0	25175	BHI medium
Streptococcus sanguinis	oral cavity	4.0	BAA-1455	ATCC medium 44, 260
Tannerella forsythia	oral cavity	4.5 (b)	43037	ATCC medium 1928, 1921
Treponema denticola	oral cavity	2.0	35405	ATCC medium 1494, 260
Veillonella parvula	oral cavity	4.0	10790	BHI medium (e)
Corynebacterium amycolatum	skin	4.3 (a)	700207	ATCC medium 44
Propionibacterium acnes	skin	2.0	11828	ATCC medium 2107, 260
Staphylococcus aureus subsp. *aureus*	skin	5.0	700699D-5 (d)	
Gardnerella vaginalis	vagina	4.5 (b)	49145D-5 (d)	
Lactobacillus crispatus	vagina	4.0 (c)	55221	ATCC medium 416
Lactobacillus gasseri	vagina	6.0	33323D-5 (d)	
Lactobacillus iners	vagina	6.2 (a)	55195	ATCC medium 1685, 260
Lactobacillus jensenii	vagina	6.2 (a)	25258D (d)	
Streptococcus agalactiae	vagina	7.0	BAA-611D-5 (d)	
