# Supplementary material for: Evaluation of 16S rRNA gene sequencing for species and strain-level microbiome analysis
Source: Nat Commun. 2019 Nov 6;10:5029. doi: 10.1038/s41467-019-13036-1 (PMC6834636; doi:10.1038/s41467-019-13036-1)
Supplement: Supplementary file 3 — Description of Additional Supplementary Files [file 41467_2019_13036_MOESM3_ESM.docx]

**Description of Supplementary Files**

**File Name:** Supplementary Data 1

**Description:** The relative abundance of all Bacteroides species detected in the stool microbiome of healthy athletes, when abundance is determined by A) mWGS sequencing, B) V1-V9 OTUs, C) V1-V3 OTUs. Table A shows the proportion of mWGS reads assigned to each Bacteroides species, determined by aligning mWGS reads to the RTG database. Table B shows the number of V1-V9 amplicons that could be assigned to each Bacteroides OTU. Table C shows the number of V1-V3 amplicons that could be assigned to each Bacteroides OTU. OTUs were generated by clustering sequences at a 99% identity threshold. The taxonomy of each OTU was determined by aligning each representative OTU sequence to the genomes present in the RTG database. In cases where multiple V1- V3 OTUs matched the same Bacteroides species, the respective species row in table A is duplicated.

**File Name:** Supplementary Data 2

**Description:** Further details of bacterial isolates cultured from the stool of healthy athletes. Sheets in this excel file contain the following information: Isolate Description: Details of the culture media and atmospheric conditions (where available) used for each isolate. The final column (OTU) indicates the OTU assignment for each isolate when clustering representative sequences at 99% identity; Isolate Taxonomy: The taxonomic classification of each isolate identified using BLAST; Isolate Technical Replicates: Details of isolates for which one or more technical replicate was available. Nanomolar (nM) loading concentrations are provided for each technical replicate; Isolate Sequencing Depths: The number of filtered amplicon sequences available for each isolate following the pooling of technical replicates; Isolate Substitutions: The number and location of substitutions detected in isolate sequences when aligning them to a single reference sequence using Cross_match. Columns denote base position within the 16S gene; Isolated Filtered Substitutions: The number and location of substitutions assumed to represent true single nucleotide polymorphisms (SNPs) within the 16S gene.
