# Supplementary material for: Evaluation of 16S rRNA gene sequencing for species and strain-level microbiome analysis
Source: Nat Commun. 2019 Nov 6;10:5029. doi: 10.1038/s41467-019-13036-1 (PMC6834636; doi:10.1038/s41467-019-13036-1)
Supplement: Supplementary file 6 — Reporting Summary [file 41467_2019_13036_MOESM6_ESM.pdf]

nature research

Corresponding author(s): Jethro Johnson

Last updated by author(s): Oct 4, 2019

Reporting Summary

Nature Research wishes to improve the reproducibility of the work that we publish. This form provides structure for consistency and transparency in reporting. For further information on Nature Research policies, see Authors & Referees and the Editorial Policy Checklist .

Statistics

For all statistical analyses, confirm that the following items are present in the figure legend, table legend, main text, or Methods section.

n/a	Confirmed	
☐	☒	The exact sample size (*n*) for each experimental group/condition, given as a discrete number and unit of measurement
☐	☒	A statement on whether measurements were taken from distinct samples or whether the same sample was measured repeatedly
☐	☒	The statistical test(s) used AND whether they are one- or two-sided<br>*Only common tests should be described solely by name; describe more complex techniques in the Methods section.*
☒	☐	A description of all covariates tested
☒	☐	A description of any assumptions or corrections, such as tests of normality and adjustment for multiple comparisons
☒	☐	A full description of the statistical parameters including central tendency (e.g. means) or other basic estimates (e.g. regression coefficient) AND variation (e.g. standard deviation) or associated estimates of uncertainty (e.g. confidence intervals)
☒	☐	For null hypothesis testing, the test statistic (e.g. *F*, *t*, *r*) with confidence intervals, effect sizes, degrees of freedom and *P* value noted<br>*Give P values as exact values whenever suitable.*
☒	☐	For Bayesian analysis, information on the choice of priors and Markov chain Monte Carlo settings
☒	☐	For hierarchical and complex designs, identification of the appropriate level for tests and full reporting of outcomes
☒	☐	Estimates of effect sizes (e.g. Cohen's *d*, Pearson's *r*), indicating how they were calculated

Our web collection on statistics for biologists contains articles on many of the points above.

Software and code

Policy information about availability of computer code

Data collection	Details of all open source and commercial computer code used is given in the text. Where appropriate, software is also listed dependencies in the custom code described below.
Data analysis	All the custom code used for analyses presented in this manuscript has been made publicly available at https://github.com/TheJacksonLaboratory/weinstock_full_length_16s

For manuscripts utilizing custom algorithms or software that are central to the research but not yet described in published literature, software must be made available to editors/reviewers. We strongly encourage code deposition in a community repository (e.g. GitHub). See the Nature Research guidelines for submitting code & software for further information.

Data

Policy information about availability of data

All manuscripts must include a data availability statement. This statement should provide the following information, where applicable:

- Accession codes, unique identifiers, or web links for publicly available datasets
- A list of figures that have associated raw data
- A description of any restrictions on data availability

Sequence data have been submitted to the NCBI Short Read Archive (BioProject IDs PRJNA552603, PRJNA305507, PRJNA561528). Other raw data used in figure generation is either already publicly available (in the case of in silico analyses), or has been made available as supplementary materials.

Field-specific reporting

Please select the one below that is the best fit for your research. If you are not sure, read the appropriate sections before making your selection.

Life sciences study design

All studies must disclose on these points even when the disclosure is negative.

Sample size	No sample size calculations were performed because this study does not report formal statistical tests.
Data exclusions	No data were excluded from this analysis unless due to quality control filtering, which is described in detail in the methods section of the text.
Replication	Technical replicates were used to establish the reliability of sequencing platforms. No biological replicates were included because this study does not report formal statistical tests.
Randomization	Randomization was not performed because it was not required by the study design.
Blinding	Randomization was not performed because it was not required by the study design.

Reporting for specific materials, systems and methods

We require information from authors about some types of materials, experimental systems and methods used in many studies. Here, indicate whether each material, system or method listed is relevant to your study. If you are not sure if a list item applies to your research, read the appropriate section before selecting a response.

Materials & experimental systems

n/a	Involved in the study	
☒	☐	Antibodies
☒	☐	Eukaryotic cell lines
☒	☐	Palaeontology
☒	☐	Animals and other organisms
☐	☒	Human research participants
☒	☐	Clinical data

Methods

n/a	Involved in the study	
☒	☐	ChIP-seq
☒	☐	Flow cytometry
☒	☐	MRI-based neuroimaging

Human research participants

Policy information about studies involving human research participants

Population characteristics	Human donors for gut microbiome samples were recruited as part of a previously published study (DOI: 10.1186/s40168-017-0320-4), full details of this population have therefore been previously published. Details relating to informed consent and handling of microbiome samples have now been added to this manuscript.
Recruitment	Human volunteers were recruited as part of a different study. As this was a methodological study focused on bacterial 16S rRNA gene, self-selection was not an issue that would have affected results.
Ethics oversight	Ethics oversight was provided by the Jackson Laboratory for Genomic Medicine (IRB#'s 1503000013 and 16-JGM-07).

Note that full information on the approval of the study protocol must also be provided in the manuscript.
